# Socio-economic inequalities in dietary intake in Chile: a systematic review

**DOI:** 10.1017/S1368980021002937

**Published:** 2022-07

**Authors:** María Jesús Vega-Salas, Paola Caro, Laura Johnson, Angeliki Papadaki

**Affiliations:** 1 Centre for Exercise, Nutrition and Health Sciences, School for Policy Studies, University of Bristol, 8 Priory Road, Bristol BS8 1TZ, UK; 2 School for Policy Studies, University of Bristol, Bristol, UK

**Keywords:** Dietary intakes, Socio-economic inequalities, Obesity inequalities, Systematic review

## Abstract

**Objective:**

Understanding the socio-economic inequalities in dietary intake is crucial when addressing the socio-economic gradient in obesity rates and non-communicable diseases. We aimed to systematically assess the association between socio-economic position (SEP) and dietary intake in Chile.

**Design:**

We searched for peer-reviewed and grey literature from inception until 31 December 2019 in PubMed, Scopus, PsycINFO, Web of Sciences and LILACS databases. Observational studies published in English and Spanish, reporting the comparison of at least one dietary factor between at least two groups of different SEP in the general Chilean population, were selected. Two researchers independently conducted data searches, screening and extraction and assessed study quality using an adaptation of the Newcastle Ottawa Quality Assessment Scale.

**Results:**

Twenty-one articles (from eighteen studies) were included. Study quality was considered low, medium and high for 24, 52 and 24 % of articles, respectively. Moderate-to-large associations indicated lower intake of fruit and vegetables, dairy products and fish/seafood and higher pulses consumption among adults of lower SEP. Variable evidence of association was found for energy intake and macronutrients, in both children and adults.

**Conclusions:**

Our findings highlight some socio-economic inequalities in diets in Chile, evidencing an overall less healthy food consumption among the lower SEP groups. New policies to reduce these inequalities should tackle the unequal distribution of factors affecting healthy eating among the lower SEP groups. These findings also provide important insights for developing strategies to reduce dietary inequalities in Chile and other countries that have undergone similar nutritional transitions.

Obesity and suboptimal diets are important risk factors for non-communicable diseases. Globally, 11 million deaths were attributed to dietary risk factors in 2017^(1)^. Extensive research has identified strong associations between socio-economic position (SEP) and health outcomes, resulting in poorer health, higher mortality and shorter life expectancy among the lower SEP groups^(2,3)^. Socio-economic inequalities in health behaviours, including diet and physical activity (PA), are major contributors to inequalities in obesity, non-communicable diseases and mortality rates^(4,5)^, making the need to address these imperative.

Chile has the third-highest obesity rate among the Organization for Economic Co-operation and Development countries^(6)^. Approximately 35 % of people aged >15 years and 25 % of 5–6-year-old children in Chile are living with obesity^(7,8)^, constituting the top risk factor for death and disability^(9)^. In addition, obesity and its related comorbidities are a burden to the Chilean economy, accounting for 2·3 % of the annual total health expenditure^(10)^.

Obesity rates vary in Chile, with higher rates reported among women and socio-economically disadvantaged groups^(11,12)^. The 2017 Chilean National Health Survey (ENS) estimated that in adults aged ≥15 years, obesity prevalence was 46·6 % *v*. 29·5 % among those with lower compared with higher educational backgrounds, respectively^(7)^. Similar socio-economic inequalities were observed in children aged 0–9 years (17·1 % *v*. 9·7 %, in lower- *v*. higher-income households, respectively)^(13)^. As obesity is caused by a long-term positive energy imbalance^(14)^, these socio-economic differences imply differences in dietary intakes and/or PA between the different socio-economic groups^(15)^. However, the causes of the unequal socio-economic distribution of diet and PA are multifactorial at the societal, community, environmental and individual levels^(16,17)^. Systematic reviews have suggested that weight gain and elevated adiposity are positively related to energy-dense diets^(18,19)^, diets relatively high in fat and sugar and low in fibre^(20)^, low in fruit and vegetable intake^(21,22)^ and high in sugar-sweetened beverage (SSB) consumption^(23,24)^.

Three reviews have examined the association between SEP and the dietary determinants of obesity in developed-western countries^(15,25,26)^. Associations between SEP and energy intake were inconsistent, but more unhealthy dietary patterns (lower in fruits, vegetables and fibre) were observed among adults^(15,25)^ and adolescents^(26)^ from lower SEP groups. However, none of these reviews included studies from South America, which would be important to establish the role of SEP in dietary intake in countries where obesity prevalence has rapidly increased in recent decades^(27)^. Also, no systematic review to date has investigated the socio-economic inequalities in diet in Chile, and it is unclear if similar trends to these earlier reviews^(15,25,26)^ would be observed in this country, which experienced a rapid transition from being a low-and-middle-income country to a high-income country since 2013^(28)^. Two other reviews from studies in low-and-middle-income countries reported a lower dietary quality (low in fat, fibre, fruits, vegetables and fish intakes) among the lower SEP groups^(29,30)^. These reviews included studies conducted in a few South American countries, but not Chilean data, because Chile had already transitioned to being a high-income country. The rapid economic and nutritional transition that Chile has experienced is similar to other parts of the world, including other Latin American, Asian, Middle Eastern and African countries^(31)^. Thus, examining the role of SEP in dietary intake in Chile throughout time will not only inform policies around dietary inequalities in this country but will also provide important insights for the development of such strategies in countries undergoing similar economic and/or nutrition transitions. The aim of the current study was, therefore, to systematically assess the socio-economic inequalities in dietary intake, specifically in the Chilean population. If available, a comparison between studies conducted in different stages of the Chilean nutritional transition and studies comparing SEP inequalities in diet in Chileans of different body weight status, age group or gender will be performed.

## Methods

**Fig. 1:**
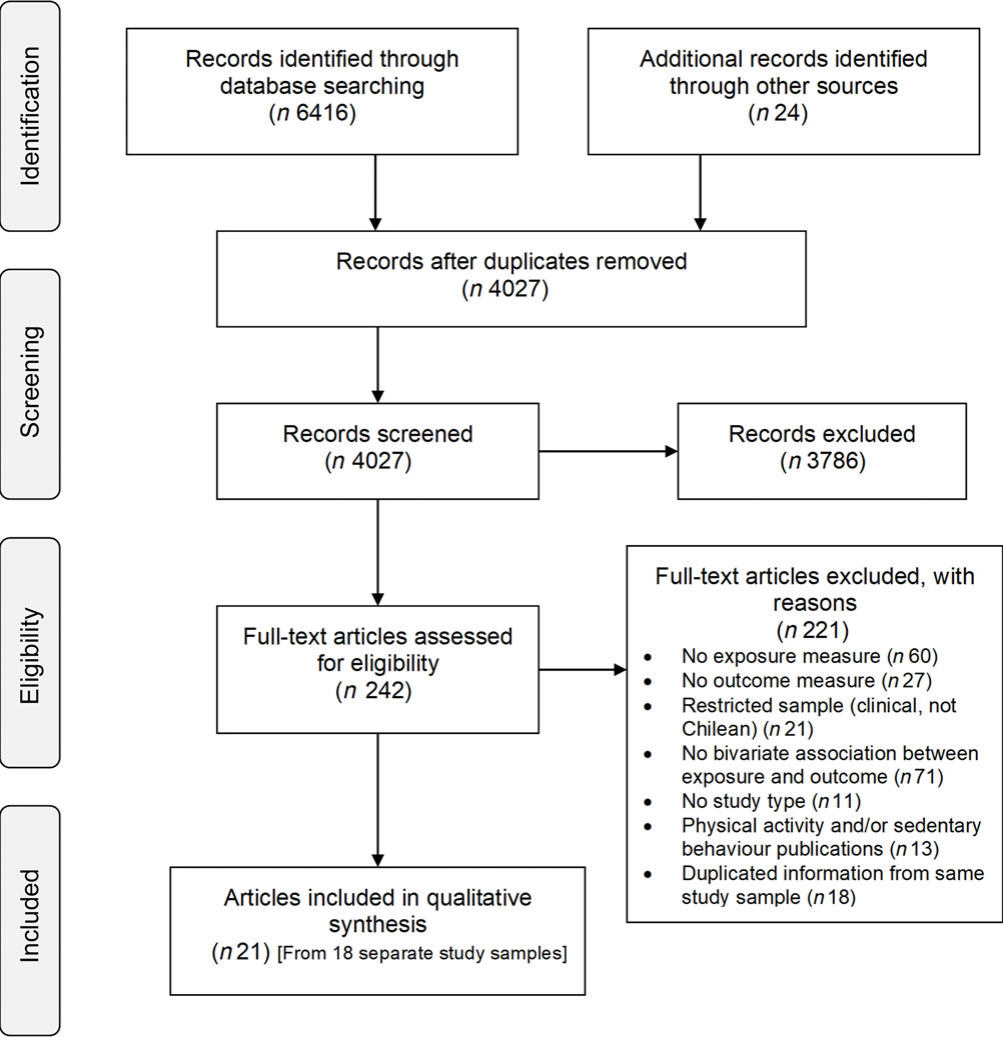
Preferred Reporting Items for Systematic reviews and Meta-Analyses (PRISMA) flow diagram of literature search and study selection

This systematic review was registered in PROSPERO (CRD42018096925) and conducted following the Preferred Reporting Items for Systematic Reviews and Meta-Analyses Equity extension (PRISMA-E 2012)^(32)^ (Fig. 1 and online supplementary material, Supplemental Table 1).

### Search strategy

Peer-reviewed and grey literature were searched in MEDLINE, Scopus, PsycINFO, Web of Science, Latin American and Caribbean Health Sciences Literature (LILACS), OpenGrey database, and national and international organisations’ websites (e.g. Chilean Ministries and Funding Organisations, WHO) (online supplementary material, Supplemental Tables 2–7). Publications from inception to 31 December 2019 were included. Searches were not date restricted as one of the secondary aims of this study was to compare studies conducted during different stages of the nutritional transition. Searches were conducted by two researchers independently (M.J.V.-S. and P.C.). Reference lists of included articles were hand-searched for additional original publications.

### Inclusion criteria

Observational studies with a cross-sectional or longitudinal design and published in English and Spanish were included. Articles were included if they reported differences of at least one indicator of dietary intake, PA or sedentary behaviour, between two or more groups of different SEP. The results for PA and sedentary behaviour will be published separately. Indicators of dietary intake were included if they were presented as quantity consumed (e.g. g/d), frequency of consumption, compliance with recommendations and/or adherence to a dietary pattern. Only studies conducted in Chile among the general population, independent of participants’ age and including information from women and men, were included. Studies conducted in clinical settings, focusing on chronic diseases that may impact on weight (e.g. diabetes, cancer, HIV), with samples sizes <100 participants and clinical trials (except if providing baseline measures of interest), were excluded.

### Title screening and selection

Title and abstract screening and full-text selection were checked against the inclusion criteria by two independent reviewers, both fluent English and Spanish speakers (M.J.V.-S. and P.C.). A good agreement between the reviewers (*κ* = 0·62)^(33)^ was obtained for a pilot test of the first 100 records. After resolving discrepancies in the pilot test, both reviewers screened the remaining titles and abstracts independently, obtaining an excellent interrater agreement (*κ* = 0·93). Discrepancies were resolved through discussion or third-party adjudication including the remaining reviewers (L.J. and A.P.). Authors were contacted if clarification on any aspect of a study was required.

If multiple publications of the same study reporting the same dietary and SEP indicators were eligible, the publication with the most complete data for the purposes of the current review was considered the primary source. In contrast, if multiple publications reported different indicators from the same study, each publication was included in the review.

### Data extraction

Data were extracted in a piloted table (online supplementary material, Supplemental Table 8). When a study reported multiple SEP or dietary factors, data extraction was conducted individually for each one. To reduce bias from the variability of confounders and mediators in the selected papers and allow comparisons across studies, we extracted unadjusted associations between each dietary indicator and at least two SEP groups.

### Outcomes

#### Dietary factors

Dietary factors of interest were ones previously related to weight gain and/or obesity^(34,35)^ and measured by at least one dietary assessment method. Due to diverse methods of reporting dietary intake between the articles, data were summarised as: (1) energy intake and macronutrients; (2) foods and food groups; (3) dietary patterns and (4) meal patterns.

#### Socio-economic position

SEP, a widely acknowledged concept that stratifies health opportunities and outcomes^(36,37)^, reflects the position of individuals or groups within a society, according to socially derived economic factors^(38)^. SEP is a relative construct and exhibits one’s place within a social hierarchy, based on (a) differential access to the actual capital or resources and (b) social status based on prestige^(39,40)^. Articles were included in this review if they considered SEP indicators either at the individual or household level, based on education, occupation and/or income, or a combination between them (composite indices). Articles reporting only area-level SEP indicators (e.g. borough or municipality) or institutional-based (e.g. school type attendance) were not included. Area-level SEP indicators are usually an aggregated measure from individual-level indicators or administrative information^(41)^, not reported directly from participants. As this study focused on comparisons of individual-level dietary intakes, only individual-level SEP measurements reported directly from participants were included. For comparison and analysis purposes, the low SEP indicator was compared against the middle to high SEP indicator.

### Quality assessment and risk of bias

Assessment of the quality of individual articles was undertaken by two reviewers independently (M.J.V.-S. and P.C.). Each study was evaluated using an adaptation of the Newcastle Ottawa Quality Assessment Scale for cohort and cross-sectional studies^(42)^ (max. 10 points) (online supplementary material, Supplemental Table 9).

### Data analysis

The magnitude of relative dietary differences between high and low SEP groups was estimated by calculating either relative differences between intakes (e.g. kcal/d) or OR for proportions (*ρ*, e.g. % of participants who consume fruit daily). Calculations were conducted using the following formulas used by previous systematic reviews in this topic^(15,29)^:
(1)

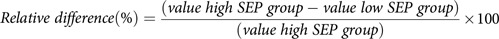



(2)

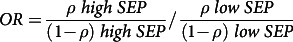




Associations were categorised according to the magnitude of the relative difference in dietary intake between the SEP groups as no association (<10 % or OR = 0·91–1·0), moderate (10–20 % or OR = 0·80–0·90) and large (>20 % or OR <0·80)^(13,25)^. Results were presented in tables (online supplementary material, Supplemental Tables 11–17) and synthesised in harvest plots^(43)^, stratifying by population age (children and adults) and quality score (three groups).

## Results

### Included articles

**Fig. 2:**
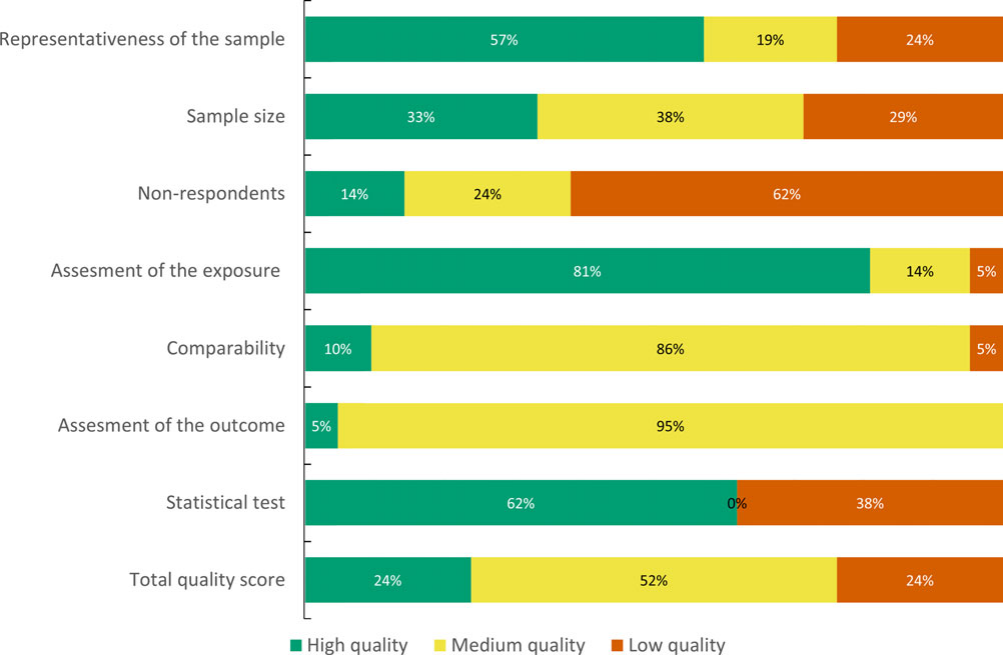
NOQAS quality assessment of included publications (N = 21) NOQAS: Newcastle Ottawa Quality Assessment Scale

The search and study selection processes are illustrated in Fig. 1. Of 4028 unique records, 242 full-text articles were assessed for eligibility and twenty-article articles (representing eighteen separate studies) were included. Only two articles collected data during the nutritional transition (1960–1989)^(44,45)^, and the remaining studies were conducted from 2000 onwards, at a post-transitional stage. The characteristics of the twenty-one articles are displayed in Table 1, and a summary of the quality assessment is presented in Fig. 2 and online supplementary material, Supplemental Table 10.

**Table 1:** Study characteristics

Author	Study name/year data collection	Location	Study design	Sample population	Sample size (*n*)	Response rate	Age group	SEP indicator	Dietary assessment method	Quality score
Adjemian *et al.*, (2007)^(49)^	N/R	Santiago	C	Children	239	N/R	7–9 years	Household index: 2 groups (low *v*. high)	2 × 24-h recall	3·5
Correa-Burrows *et al.*, (2015)^(61)^	SIMCE 2009	Santiago	C	Children	1074	84 %	9–10 year/13–14 years	Household index (Graffar’s modified scale): 3 groups (high and medium-high *v*. medium-low; low)	FFQ	6·5
Essman *et al.*, (2018)^(56)^	FEChiC 2016	Santiago	L	Children	961	N/R	3–5 years	Mother’s education level: 3 groups (less than high school or lower *v*. more than high school)	24-h recall	6·5
	GOCS 2016	Santiago	L	Adolescents	768	N/R	12–14 years			
Hoffmeister *et al.*, (2016)^(55)^	2009–2010	Bio-Bío, Araucanía, Los Lagos-Los Ríos, and Aysén- Magallanes	C	Children	2987	N/R	2 years and 4 years	Household index: 3 groups (low *v*. high)	FFQ	4·5
Ivanovic *et al.*, (1991)^(44)^	1982	Santiago	C	Children	550	N/R	13–16 years	Household index: 3 groups (low *v*. high)	24-h recall	5·5
Ivanovic *et al.*, (1992)^(45)^	1986–1987–1989	Santiago	L	Children	488	75 %	5–18 years	Household index (Graffar’s modified scale): 3 groups (low *v*. high + medium-high)	24-h recall	5
Jensen *et al.*, (2019)^(63)^	FEChiC 2016	Santiago	L	Children	961	N/R	4–6 years	Mother’s education level: 3 groups (less than high school or lower *v*. more than high school)	24-h recall	6·5
	GOCS 2016	Santiago	L	Adolescents	768	N/R	12–14 years			
Liberona *et al.*, (2011)^(47)^	2007	Santiago	C	Children	1732	96 %	9–12 years	Household index (ESOMAR): 4 groups (middle-low + low *v*. very high + high)	24-h recall	6
Cediel *et al.*, (2018)^(53)^	ENCA 2010–2011	Chile	C	Children and adults	4920	85 %	≥2 years	Breadwinner educational level: 3 groups (≤8 years of education *v*. ≥12 years of education) Family income (1 *v*. ≥6 minimum wages)	24-h recall	7·5
Celis-Morales *et al.*, (2011)^(46)^	GENADIO 2008	Santiago, Los Rios, Bio-Bio	L	Adults	472	54 %	20–60 years	Household index (ESOMAR): 3 groups (low *v*. high) Educational level: 3 groups (primary *v*. tertiary)	7-d food diary	5
Duran-Aguero *et al.*, (2015)^(57)^	2014	Santiago, Temuco, Viña del Mar, Concepción and Antofagasta	C	Adults	486	190 %	≥18 years	Household index (ESOMAR): 3 groups (*v*. low *v*. high)	FFQ	4·5
Echeverria *et al.*, (2016)^(59)^	IDM-Chile 2010–2014	Chile	C	Adults	53 366	N/R	≥20 years	2 groups (≤12 years *v*. >12 years)	FFQ	2
Fisberg *et al.*, (2018)^(50)^	ELANS 2014–2015	Chile	C	Adults	879	N/R	15–65 years	Household index (AIM): 5 groups (low *v*. high)	2 × 24-h recall	6
Gomez *et al.*, (2019)^(60)^	ELANS 2014–2015	Chile	C	Adults	879	N/R	15–65 years	Household index: 3 groups (low *v*. high)	2 × 24-h recall	6
Ministerio de Salud de Chile^(54)^	ENCAVI 2006	Chile	C	Adults	6210	98 %	≥15 years	Income quintile: 5 groups (1st quintile *v*. 5th quintile)	FFQ	7
Ministerio de Salud de Chile^(62)^	ENETS 2009–2010	Chile	C	Adults	9503	74 %	≥15 years	Education: 7 groups (Incomplete primary *v*. complete university) Income level: 6 groups (<$136·000 *v*. >$851·000 CLP) Employment status: 2 groups (non-occupied *v*. occupied) Employment situation: 6 groups (dependent worker *v*. owner)	FFQ	7
Ministerio de Salud de Chile^(51)^	ENS 2009–2010	Chile	C	Adults	5434	85 %	≥15 years	Education: 3 groups (<8 years *v*. >12 years)	FFQ	9
Ministerio de Salud de Chile^(48)^	ENCA 2010	Chile	C	Children and adults	4920	86 %	≥2 years	Household index (AIM): 5 groups (low *v*. high)	FFQ & 24-h recall	8·5
Ministerio de Salud de Chile^(7)^	ENS 2016–2017	Chile	C	Adults	6233	90 %	≥15 years	Education: 3 groups (<8 years *v*. >12 years)	FFQ	6
Pinto *et al.*, (2019)^(58)^	ELANS 2014–2015	Chile	C	Adults	879	N/R	15–65 years	Household index: 3 groups (low *v*. high)	2 × 24-h recall	4·5
Ratner *et al.*, (2008)^(52)^	N/R	Santiago and Valparaíso	C	Adults	1745	58 %	≥18 years	Education: 2 groups (up-to-secondary *v*. vocational/university)	FFQ	4

N/R, not reported; SEP, socio-economic position; C, cross-sectional; L, longitudinal; SIMCE, system for the assessment of educational quality test; ENCAVI, Chilean National Quality of Life and Health Survey; ENS, Chilean National Health Survey; ENETS, Chilean workers employment conditions, work, health and quality of life survey; ENCA, Chilean National Food Intake Survey; IDM-Chile, Chilean Mediterranean Diet Index; ELANS, Latin American Study of Nutrition and Health; AIM, Chilean Marketing Research Association; ESOMAR, World association of market research.

() Lower and higher SEP group compared.

### Energy and macronutrients

Most of the articles used a 24-h recall as the dietary assessment method. These articles utilised composite indices to assess SEP, except for one study that used a 7-d food diary and reported on two SEP indicators: education and SEP index^(46)^. Overall, the gathered evidence shows a variable evidence of association between energy and macronutrient intake and SEP among children and adults.

#### Energy intake

**Fig. 3:**
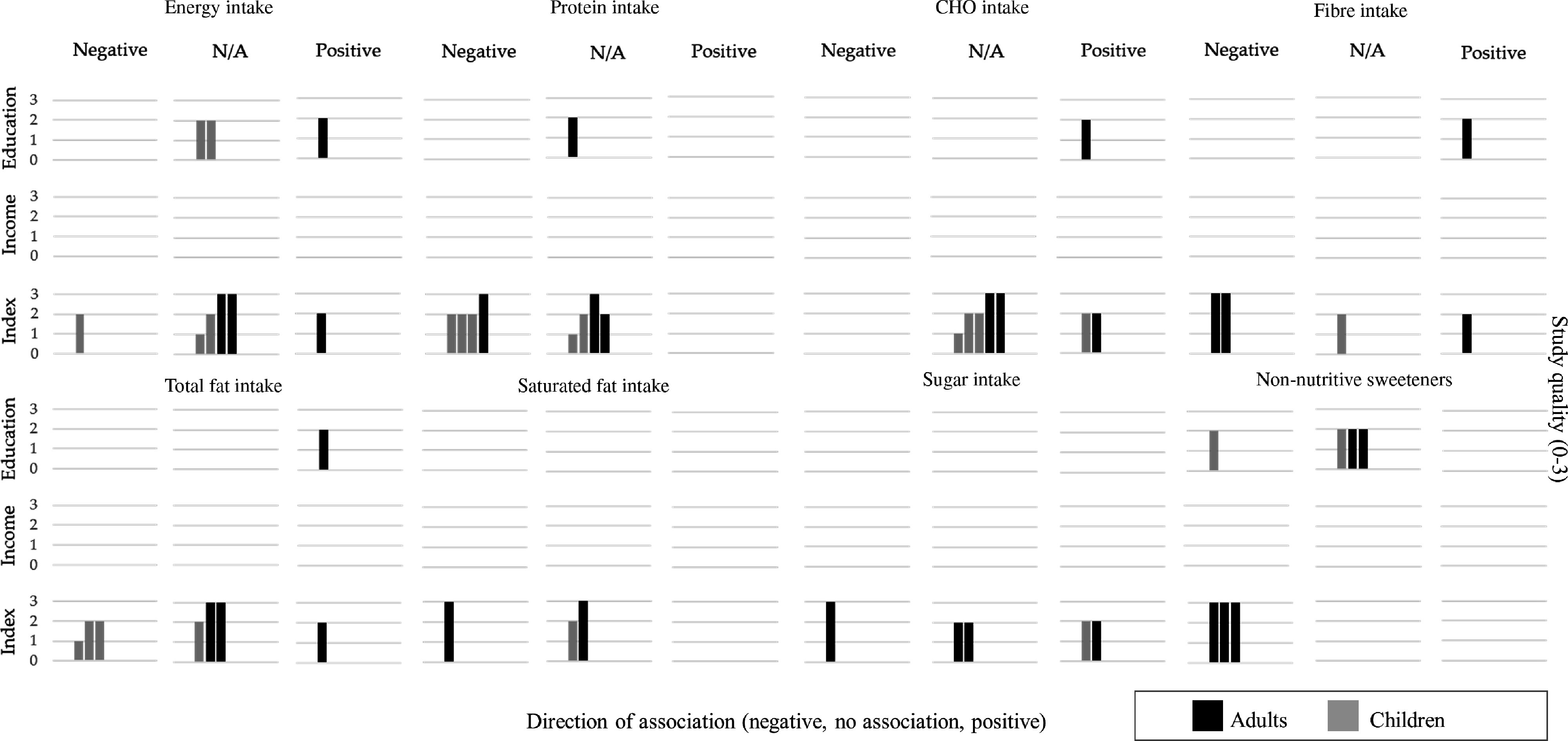
Summary of evidence for associations between socioeconomic position and dietary intakes – Energy intake, macronutrients and non-nutritive sweeteners Each row represents a dimension of socioeconomic position, and each column represents the direction of the association between socioeconomic position indicators and dietary intakes. Relative differences =10 % or OR < 0.80 were categorised as negative association (lower intakes among lower SEP groups, compared to the higher) or positive association (higher intakes among lower SEP groups, compared to the higher). Relative differences <10 % were classified as no association (N/A). Each bar represents an association between SES and dietary intakes. The quality assessment scores from the articles are indicated by the height of the bars (1 = Quality scores = 4.5; 2 = Q.S. > 4.5 and < 7; 3 = Q.S. = 7). Studies conducted among children population are presented with half-tone (grey) bars and studies conducted among adults are indicated with full-tone (black) bars.

Six articles, with four of them focusing on schoolchildren, examined a total of nine associations between energy intake and indicators of SEP (Fig. 3 and online supplemental material, Supplemental Table 11). Among the five associations reported for children, only one moderate association from a study conducted during the 1980s was reported for lower energy intake among the lower SEP group, compared with the higher (Δ = −17·2 %)^(44)^. The GENADIO study, a medium-quality study focused on adults and indigenous populations, reported two large associations for higher energy intake among the lower SEP group (relative difference (Δ) = 20·6 % and 22·1 % of kcal/d). All remaining associations among children and adults reported no associations (<10 %).

#### Protein

Six articles examined nine different associations between protein intake and SEP (Fig. 3 and online supplementary material, Supplemental Table 11). Three out of five associations reported for children suggested a lower protein intake among children from the lower SEP group, expressed either as a percentage of nutrient adequacy intake (Δ = −37·7 and −13 %)^(44,45)^ in studies conducted in the 1980s or g/d (Δ = −15 %)^(47)^. One study conducted among adults reported differences only for women, with lower protein intakes among the lower SEP groups (Δ = −10·4 %)^(48)^. The remaining five associations among adults and children reported no associations (<10 %).

#### Carbohydrates

Six articles reported on eight different associations between carbohydrates and SEP (Fig. 3 and online supplementary material, Supplemental Table 11). One study conducted during the 1980s out of the four articles assessing children’s intakes reported a moderate higher intake of carbohydrates (as % of total energy intake) among the lowest SEP (Δ = 17·3 %)^(44)^. Among adults, only the GENADIO study reported a moderate higher carbohydrate intake among the lower SEP groups (Δ = 16·8 and 18·4 %). The remaining two associations reported no associations (<10 %).

#### Fat

Six articles examined eight different associations (four in children) between total fat intake and SEP and three associations between saturated fat and SEP (one in children) (Fig. 3 and online supplementary material, Supplemental Table 12). Three out of the four associations among children reported a consistent lower total fat intake among the lower SEP group, either measured as g/d (Δ = −10·9 %)^(49)^ or % of total energy intake in studies conducted in the 1980s (Δ = −13·3 and −25·5%)^(44,45)^. Among adults, only the GENADIO study reported larger differences for adults, with higher total fat intake among the lower SEP group (Δ = 29·0 and 37·2%). Regarding saturated fat intake, only one out of the three associations suggested a relevant lower saturated fat intake among the lower SEP (Δ = −16·3% among adult women)^(48)^.

#### Fibre

Three articles reported five associations between dietary fibre and SEP, with inconsistent associations ranging from Δ = −12 to 59·6 % (Fig. 3 and online supplementary material, Supplemental Table 12). One study among children did not report any major differences between the SEP groups.

#### Sugar

Three articles, one in children and two in adults, reported four associations between sugar intake and SEP (Fig. 3 and online supplementary material, Supplemental Table 12) with inconsistent results (Δ = −16·5 to 35·2 %). When expressed as grams of daily intake, children from the lower SEP groups presented a higher sugar intake (Δ = 35·2 %)^(47)^, but results among adults were inconsistent (Δ = −16·5 and 11·4 %)^(48,50)^.

### Food groups

Most articles used FFQ to assess food group intakes and focused only on adult populations. Overall, findings indicate a strong evidence for moderate-to-large associations between lower intake of fruit and vegetables, dairy products and fish/seafood and higher pulses consumption among adults of lower SEP.

#### Fruits and vegetables

**Fig. 4:**
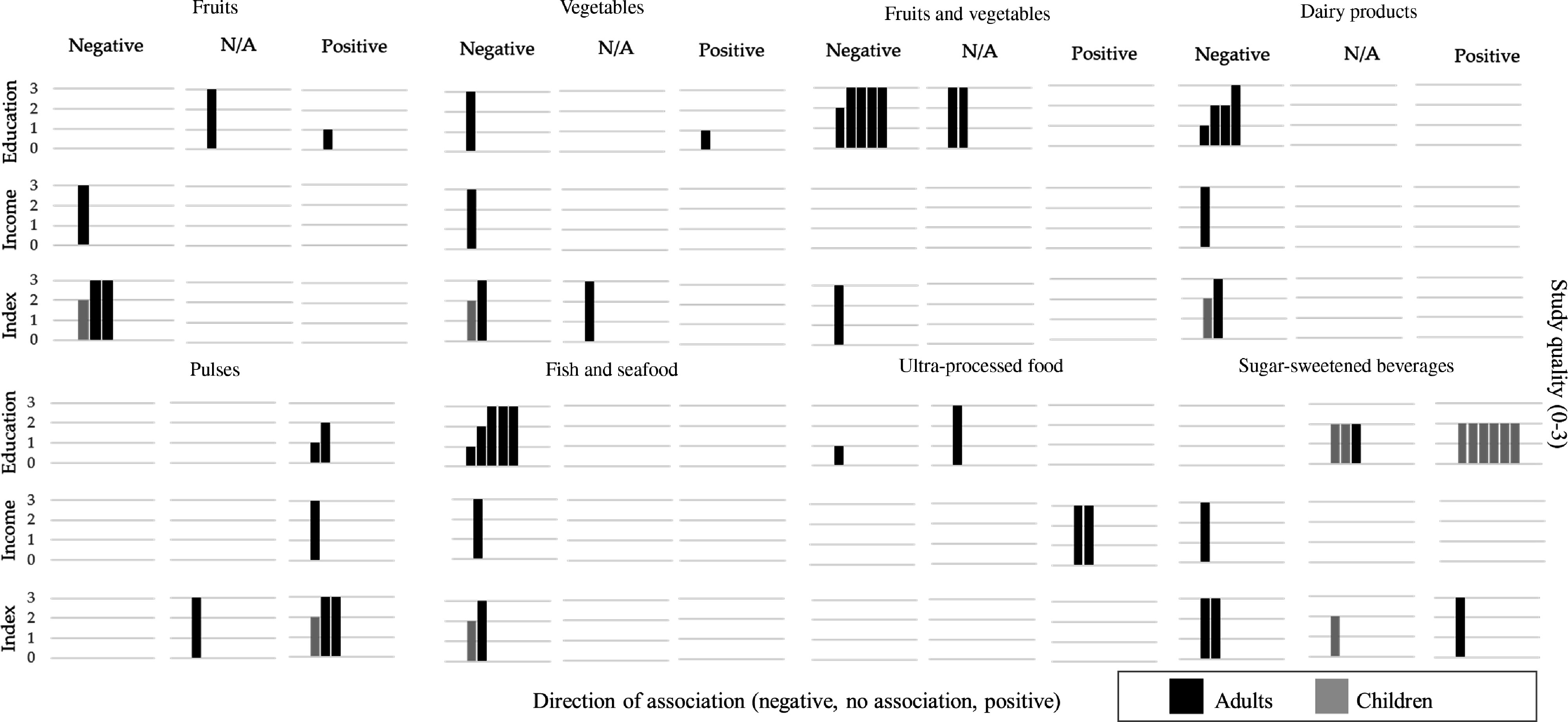
Summary of evidence for associations between socioeconomic position and dietary intakes – Food groups Each row represents a dimension of socioeconomic position, and each column represents the direction of the association between socioeconomic position indicators and dietary intakes. Relative differences =10 % or OR < 0.80 were categorised as negative association (lower intakes among lower SEP groups, compared to the higher) or positive association (higher intakes among lower SEP groups, compared to the higher). Relative differences <10 % were classified as no association (N/A). Each bar represents an association between SES and dietary intakes. The quality assessment scores from the articles are indicated by the height of the bars (1 = Quality scores = 4.5; 2 = Q.S. > 4.5 and < 7; 3 = Q.S. = 7). Studies conducted among children population are presented with half-tone (grey) bars and studies conducted among adults are indicated with full-tone (black) bars.

The associations between fruit, vegetable and combined fruit and vegetable intake and SEP are presented in Fig. 4 and online supplementary material, Supplementary Table 13. Five articles among adult populations assessed twelve associations for fruit and vegetable intake separately. Overall, most studies presented a strong case for lower consumption of fruit and vegetables among the lower SEP groups. Expressed as g/d, the difference equated to a 20–30 % lower fruit and vegetable intake among SEP adults and children (Δ = −31·1 and −20·5 % for adults and −23·9 and −31·8 % for children, respectively)^(47,48)^. Three large-national surveys reported on eight associations between fruit and vegetables (combined) and SEP^(7,48,51)^. All reported associations consistently showed a small-to-moderate lower odds of consuming ≥5 portions of fruit and vegetable a day among the lower SEP groups (OR between 0·47 and 0·81). Larger SEP differences were reported among women when fruit and vegetable intake was expressed as g/d and % of participants consuming ≥5 portions of fruit or vegetables per day (Δ = −16·8 % women *v*. −0·8 % men and OR = 0·73 *v*. 0·84, respectively)^(51)^.

#### Dairy products

Five articles presented seven associations between dairy products and SEP; all indicated a lower consumption of dairy products among the lower SEP groups (Fig. 4 and online supplementary material, Supplemental Table 14), with OR between 0·41 and 0·58 for % participants meeting the guidelines of ≥3 portions daily^(7,48,51)^. Correspondingly, a study in children reported lower grams of daily dairy product intake among the lower SEP groups (Δ = −32·4 %)^(47)^.

#### Pulses

Overall, six out of the seven associations from five articles reporting associations between consumption of pulses and SEP showed that pulses consumption was higher and more frequent among the lower SEP groups when expressed as both g/d (Δ = 16·4 % in adults and 89·3 % in children)^(47,48)^ and % of participants meeting the guideline of ≥2 portions of pulses weekly (OR = 1·53 and 1·53)^(7,48)^ (Fig. 4 and online supplementary material, Supplemental Table 14).

#### Fish and seafood

Six articles reported on eight associations between fish and combined fish and seafood consumption and SEP (Fig. 4 and online supplementary material, Supplemental Table 14). All these reports indicated a lower consumption among the lower SEP groups. The magnitude of these differences was moderate-to-large, especially when expressed as % of participants meeting the guideline of consuming fish ≥2 times weekly (OR = 0·32, 0·52 and 0·83)^(7,48,52)^. Similar associations were reported for combined fish and seafood weekly consumption (OR between 0·45 and 0·60)^(51)^. Correspondingly, children from lower SEP groups reported lower g/d of fish, compared with their higher counterparts (Δ = −45·5 %)^(47)^.

#### Wholegrains

Only one study reported on three associations on the frequency of consumption of wholegrains, showing a considerably lower consumption among the lower SEP adults, men and women (OR between 0·25 and 0·27)^(51)^ (online supplementary material, Supplemental Table 15).

#### Ultra-processed and fried foods

Three articles assessed four associations between ultra-processed and fried foods and SEP, with inconsistent results (Fig. 4 and online supplementary material, Supplemental Table 15). One study using the high-quality ENCA survey reported a moderate–higher consumption of this food group among the higher income groups, compared with the lowest, but no major difference when using education (Δ = 21·4 and 5 %, respectively)^(53)^. The two remaining associations over fried food consumption were inconsistent in direction (OR = 1·11 and 0·81, respectively)^(52,54)^.

#### Sugar-sweetened beverages

Fourteen associations (nine in children) from six articles showed inconsistent findings for the association between SSB and SEP (Fig. 4 and online supplementary material, Supplemental Table 15). One study focusing on children aged 2 and 4 years reported a more frequent intake among children from lower SEP families (OR between 1·45 and 2·74)^(55)^. In contrast, another study in children aged 3–5 years reported no major differences between SEP groups (Δ = −6·4 and 0·8 %), whereas, among adolescents aged 12–14 years, moderate differences were reported for calories/d and % from total energy intake from beverages high in sugar, calories, fat or sodium (17·2 and 11·5 %, respectively)^(56)^. Adults from the lower SEP groups displayed a more frequent consumption of SSB, but a lower amount when intake was expressed as ml/d and grams of sugar from beverages (OR = 1·61 and Δ = −32·9 and −28·8 %, respectively)^(48)^.

#### Non-nutritive sweetened beverages and products

Three articles reported on seven associations between non-nutritive sweetened beverages and products, and SEP (Fig. 4 and online supplementary material, Supplemental Table 16). A high-quality national survey reported a lower intake of these beverages, expressed both as ml/d and as frequency of consumption (Δ = −46 % and OR = 0·11, respectively) and a less frequent consumption of non-caloric sweeteners (OR = 0·28) among adults from the lower SEP^(48)^. A study conducted among university students did not report major differences^(57)^. A lower moderate % of total energy intake from beverages low in calories/sugar/fat/Na was reported among lower SEP adolescents aged 12–14 years (Δ = −11·5 %), whereas no major differences were reported for among children aged 3–5 years^(56)^.

### Dietary patterns

Five articles reported on nine associations (one in children) between a dietary pattern index and SEP (online supplementary material, Supplemental Table 16). Moderate-to-large differences were reported for lower SEP groups of adults following more unhealthy dietary patterns (e.g. low in fruits, vegetables, pulses, wholegrains, dairy and pulses, and high in trans-fat, SSB and processed meat) compared with the respective higher SEP groups (OR between 1·51 and 12·63)^(48,58)^. A moderate difference was reported for lower adherence to a Mediterranean diet and to a healthier diet (e.g. high in fruits, vegetables, pulses, wholegrains, milk and dietary fibre) among lower SEP groups (Δ = −10·9 and −12·6 %)^(59,60)^. Children from lower SEP groups reported higher chances of following a dietary pattern with high loadings for unhealthy snack foods (e.g. high in saturated fat, fibre, sugar and salt) (OR = 1·38)^(61)^.

### Meal patterns

Four articles presented fifteen associations related to meal patterns and SEP (online supplementary material, Supplemental Table 17). Overall, inconsistent results between frequency of daily breakfast consumption and SEP were reported among three large national surveys in adults^(48,54,62)^. Small-to-moderate but consistent results were reported among men from the lower SEP group, measured either by education, income or occupation, consuming ‘breakfast on the previous day’ less frequently (OR = 0·90, 0·40 and 0·86)^(62)^; inconsistent results were reported among women (OR = 2·03, 0·51 and 1·31). Two national surveys including adults reported different directions of association for everyday breakfast consumption (OR = 0·81 and 1·13)^(48,54)^. The FeChiC and GOCS studies focused on children and adolescents and did not report major differences between SEP groups in snacks or number of meals/d^(63)^.

## Discussion

To our knowledge, this is the first study to systematically assess the evidence of socio-economic inequalities in dietary intake in Chile. We found consistent evidence for poorer quality food intake among lower SEP groups, specifically, a lower consumption among low SEP groups of healthier food groups like fruits, vegetables, dairy and fish, which could underpin the socio-economic gradient in obesity.

Our findings for socio-economic gradients in energy and macronutrient intake were inconsistent across studies and among children and adults. These results are similar to previous studies in other populations reporting smaller or inconclusive associations between macronutrient intake and SEP, but larger and strong associations between SEP and food consumption^(64)^. Among children, there was some weak evidence for a moderate association between lower protein and fat intake and lower SEP groups, primarily reported in studies conducted during the 1980s^(44,45)^. Similar findings were reported by previous reviews including studies from low-and-middle-income countries^(29,30)^.These results could be capturing aspects of the rapid nutritional transition that Chile underwent between the 1960s to 1980s^(65)^. This period is characterised by increased intakes of refined carbohydrates and animal products, initially among the higher SEP groups and later, across the entire population^(66,67)^. Our findings suggesting lower SEP adults consumed a more obesogenic balance of foods and nutrients can relate to the post-transition stage that Chile is placed since the 1990s^(65)^. This period is characterised by consumption of a more ‘Western’ diet, high in saturated fat, refined carbohydrates, sugar and sodium, and low in fibre, especially among the lowest SEP groups^(68–70)^. This post-transition stage has been linked with the economic development and modernisation of the country that placed Chile among high-income countries^(28)^.

Inconsistent associations observed for energy intake and macronutrients can be linked to poor measurement, as most studies used a single 24-h recall to assess nutrient intakes. The estimation of energy intake is less accurate when using only one 24-h recall^(71)^ owing to day-to-day variability in energy intakes^(72)^, acknowledged over-reporting among children^(73)^ and variable estimations according to socio-demographic characteristics among adults and adolescents^(74–76)^. Underreporting in a Latin American study of adults was higher among women, older ages, people with low education level and people living with overweight or obesity^(77)^. Future studies should therefore account for bias introduced by dietary assessment methods.

Our findings suggesting a lower intake of fruit and vegetable, dairy product, fish and wholegrain among Chilean adults and a higher SSB consumption among children from the lower SEP are consistent with previous systematic reviews^(15,29,30,78–83)^. These results support our findings over a less healthy dietary pattern among the lower SEP groups. Healthy dietary patterns have been consistently pointed out as protective for overweight and obesity^(18,21,22,84–86)^ and CVD risk^(21,22,87–96)^. Dietary guidelines, in Chile and worldwide, recommend the consumption of these foods over more energy-dense and nutrient-poor foods^(97–100)^. However, policies tend to focus solely on restricting energy-dense nutrient-poor foods and ignore the need to promote/enable healthier foods intake. Our results reinforce the need for equity-based policy action to address the inequalities in healthy food intake.

Our review showed a higher relative intake of pulses among lower SEP groups. Pulses are considered protective against CVD and obesity^(101–103)^. Despite the consistent decrease in pulses’ purchases among the Chilean population since the 1980s, this reduction is less pronounced among the lower SEP groups^(104)^. The relatively lower price of pulses, compared with animal protein sources, offers a good nutritional value for money^(105)^ and might explain the difference among SEP groups. To our knowledge, no previous systematic review has compared pulses consumption between socio-economic groups. A methodological limitation of previous studies relates to the number of studies assessing pulses and fruits and vegetables together (i.e. in the same food group)^(106)^ or as part of a dietary pattern (e.g. Mediterranean diet)^(107)^. Studies assessing pulses together with other foods have consistently reported a lower consumption among lower SEP adolescents^(108,109)^ and adults^(110,111)^. Our novel finding places a question around the role of pulses in the social gradient of obesity in Chile. Historically, lower SEP groups in Chile consumed more pulses stews due to economic and food restrictions before the nutritional transition, and therefore, pulses are commonly considered to be ‘indigenous and poor’s food’^(112)^. Throughout the country, pulses stews are commonly prepared with pasta or rice and a meaty protein, usually sausages^(113,114)^; it might be that these other foods, consumed alongside pulses, counteract any beneficial link between pulses and obesity. Policies aiming to increase pulses consumption and/or substitute animal protein intake for pulses for health and environmental reasons^(115,116)^ should consider the social and cultural meanings attached to pulses preparation and consumption in Chile^(117)^.

The consistent overall lower dietary quality among the lower SEP groups might have several explanations. Some studies suggest a nutritional knowledge disparity between SEP groups, with people from higher SEP reporting higher levels of nutritional knowledge and awareness of the dietary recommendations^(118–120)^. Intervention studies conducted in Chile and other countries have been successful in increasing nutritional knowledge, but they tend to not lead to changes in adiposity^(121–123)^. Cost is considered one of the main structural barriers of accessing healthier diets^(110,111,124)^. A Chilean study concluded that the costs/d and per kilocalorie of a diet adhering to the dietary guidelines are higher, compared with a less healthy diet^(125)^. Chilean households from the lower-income-quintile spent about a third of their household income in food, compared with 12 % among the highest quintile^(126)^. As a result, it is expected that lower-income households will prefer more cost-effective food options, which often constitute processed foods high in carbohydrates, meats and SSB^(104)^. Further policies aiming at reducing the relative higher healthy food cost are needed.

The rapid changes introduced into the food environment during the 1980s and 1990s in Latin America due to the liberalisation and privatisation of food industries led to the establishment of large trans-national food producer firms, supermarkets and fast-food chains, reducing food prices and pushing small-scale businesses off the market^(66,127)^. However, changes in food environments have not occurred equally across SEP groups, with several reviews reporting more obesogenic food environments among the lower SEP groups^(128–131)^. A study comparing two boroughs in Chile reported a higher concentration of food outlets (convenience and liquor stores) in the poorer areas^(132)^. Nevertheless, no information regarding the quality of the food offered by these food outlets was assessed. Future research should focus on mapping the links between the food environment in Chile and individual food intake among the lower SEP groups.

Our findings highlight the importance of developing policies to tackle dietary and obesity inequalities in Chile. Large Chilean nutritional policies have focused predominantly on campaigning programs, such as ‘5-a-day’ to promote fruit and vegetable consumption^(133)^ and ‘Choose to live healthier’ to promote healthy lifestyle behaviours^(134)^. However, several studies have concluded that large nutrition educational interventions are not sufficient to induce long-term behaviour change required to impact obesity prevalence^(135,136)^. In 2016, a national policy introduced mandatory regulations for front-of-package labelling, food advertising and school-food sales restrictions aiming to modify consumers’ choices^(137)^. Our review found little evidence of a social gradient in the food groups targeted by these policies, so it is unclear if current policies will address inequalities. The introduction of a sugary drink tax in 2014 has contributed to decreasing purchases of soft drinks, albeit mostly among higher SEP groups, which brings into question the effectiveness of the tax to reduce inequalities in consumption^(138)^. Policies should avoid the potential regressive impact of unhealthy food and drink taxes and complement them with healthy food subsides^(139,140)^. Due to the interconnected determinants of dietary behaviours at individual, social, political and cultural levels, a whole-system approach^(141)^ should be implemented for reducing the inequalities in dietary behaviour by integrating policies at global, national and local levels^(17)^.

The socio-economic inequalities in dietary patterns, with more unhealthy diets being observed among the lower SEP groups, can contribute to the already examined inequalities in health and health-related outcomes^(2,4,142)^. As stated by the Social Determinants of Health framework, tackling inequalities on the structural determinants will impact on improving health, particularly among the most deprived SEP groups^(143)^. Considering the high-income inequality in Chile^(144)^, it is more urgent than ever to tackle inequalities that affect most people living in areas with low income and poorer education. These claims were also raised by the Chilean protests that began in October 2019^(145,146)^ and have become highly relevant during the Covid-19 pandemic^(147–149)^, raising awareness over food inequalities among the different SEP groups within and between countries.

To our knowledge, this is the first study to systematically examine the evidence of the socio-economic inequalities in dietary intake in Chile. We included both peer-reviewed and grey literature published in both English and Spanish, aiming to reduce publication bias. We followed transparent and rigorous methodology according to the PRISMA-E guidelines^(32)^. Also, and in contrast to earlier reviews assessing socio-economic inequalities in dietary intake in adults only^(15,29)^, this review includes data from both adults and children.

Our ability to draw robust conclusions on social gradients in dietary intake was limited by the heterogeneity of indicators measuring SEP and the wide range of dietary factors examined. Different cut-off points and operationalisations were used for dietary and SEP indicators, which may have contributed to the variation of the size of associations reported and prevented us from quantitatively pool estimated associations. Despite these inconsistencies in reporting, the current review presented evidence on all available dietary factors and SEP indicators reported in articles in the Chilean population to date. Our findings highlight the need for using homogeneous indicators and reporting multiple SEP indicators, as interpretations of differences in health-related behaviours between SEP groups can vary according to the SEP indicator used^(40)^. Further qualitative research would also be valuable to explore why and how the dietary inequalities we observed exist.

All but four articles included in our review were cross-sectional in design, limiting causality inferences and increasing potential risk of reverse causation. Further studies should assess the longitudinal effect of SEP in diet throughout the life course, including how childhood SEP can influence dietary behaviours in adulthood. Due to the high heterogeneity between the definition and measurement of dietary factors and SEP indicators across studies, we decided to extract unadjusted associations between a dietary indicator and two SEP groups. Using adjusted results might have contributed to a higher variation in the associations observed, whereas unadjusted associations are limited in accounting for residual confounding and mediation associations but allow comparisons across studies. To minimise the risks associated with unadjusted associations, we decided to use a conservative cut-off point (10 %) when assessing the magnitude and relevance of associations in our data synthesis. Nevertheless, earlier studies have suggested that modifications in energy intake as small as 1–2 % can have a long-term benefit in body weight change^(150)^.

Another potential source of heterogeneity in our findings is the population and sample size in the included articles. Articles among children and adolescents included different age groups and had a variety of sampling techniques (mostly convenience sampling). Among adults, we encountered different population groups, varying from university students^(57)^, workers^(52,62)^, and general and indigenous populations^(46)^. Future surveys assessing health and nutrition outcomes among the general population should ensure representation of the whole population and valid measurements of constructs. Studies whose quality was assessed as high risk, mostly lacked on reporting about non-respondent characteristics, had lower response rates and did not provided information about inferential statistics (CI or standard deviation, and *P*-values) for SEP group comparisons. Further studies should aim to implement strong and rigorous sample designs to reduce non-response bias and enhancing representativeness of the sample and results^(151)^.

A final limitation relates to the lack of studies conducted during the Chilean nutritional transition stage (1960–1990). Only two studies were conducted during the nutritional transition stage^(44,45)^; therefore, no meaningful comparisons could be undertaken between studies conducted during/after the transitional stage. Also, the lack of information for stratified associations between diet and SEP by gender, age and obesity status limited the comparisons for our second aim. Only two studies reported associations between dietary intake and SEP separately for women and men^(48,51)^, and none stratified by age or body weight status. Considering the higher prevalence of obesity among women from the lower SEP groups in Chile and worldwide^(152–156)^, further research looking at the SEP-gender determinants of dietary behaviour is needed. Also, only a few articles reported on dietary-SEP inequalities among children. In these articles, intakes were mainly assessed using 24-h recalls completed by parents, which might be challenging due to the multiple carers and settings involved in the daily life of a child^(157)^. Scarce information was also reported for the consumption of individual food groups among children; therefore, it was not possible to establish a clearer picture of children’s dietary patterns. As energy and macronutrient assessments do not provide sufficient evidence to understand the obesity-socio-economic gradient among children, further studies are needed to assess the dietary intake of this age group and examine its association with SEP.

## Conclusions

This review focused on synthesising, for the first time, the evidence on the socio-economic inequalities in dietary intake in the Chilean general population. Overall, we found consistent evidence that the consumption of fruit and vegetables, dairy products, wholegrains and fish and seafood was lower, and the consumption of pulses was higher, among adults from the lowest socio-economic group, compared with the highest. Likewise, lower SEP groups engaged more in less healthy dietary patterns, reinforcing the inequalities reported for the aforementioned food groups. Our review provides insights for public health researchers and policymakers aiming to tackle socio-economic inequalities in dietary intake. These findings highlight the need for more equity-based policy action to complement existing policies aiming at limiting the consumption of energy-dense nutrient-poor foods across the population.

## Supplementary material

For supplementary material accompanying this paper visit https://doi.org/10.1017/S1368980021002937. click here to view supplementary material
