# Supplementary material for: Socio-economic inequalities in dietary intake in Chile: a systematic review
Source: Public Health Nutr. 2021 Jul 12;25(7):1819–34. doi: 10.1017/S1368980021002937 (PMC9991770; doi:10.1017/S1368980021002937)
Supplement: Supplementary file 1 [file S1368980021002937sup.zip › S1368980021002937sup002.docx]

## **Supplementary material ‘Socioeconomic Inequalities in Dietary Intake in Chile: A Systematic Review’**

### *Table 1. Checklist of Items for Reporting Equity-Focused Systematic Reviews*

| **Section** | **Item** | **Standard PRISMA Item** | **Extension for Equity-Focused Reviews** | **Pg #** |
| --- | --- | --- | --- | --- |
| **Title** |  |  |  |  |
| **Title** | 1 | Identify the report as a systematic review, meta-analysis, or both. | Identify equity as a focus of the review, if relevant, using the term equity | 1 |
| **Abstract** |  |  |  |  |
| **Structured summary** | 2 | Provide a structured summary including, as applicable: background; objectives; data sources; study eligibility criteria, participants, and interventions; study appraisal and synthesis methods; results; limitations; conclusions and implications of key findings; systematic review registration number. | State research question(s) related to health equity. | 1 |
|  | 2A |  | Present results of health equity analyses (e.g. subgroup analyses or meta-regression). | 1 |
|  | 2B |  | Describe extent and limits of applicability to disadvantaged populations of interest. | 1 |
| **Introduction** |  |  |  |  |
| **Rationale** | 3 | Describe the rationale for the review in the context of what is already known. | Describe assumptions about mechanism(s) by which the intervention is assumed to have an impact on health equity. | 2 |
|  | 3A |  | Provide the logic model/analytical framework, if done, to show the pathways through which the intervention is assumed to affect health equity and how it was developed. | 2 |
| **Objectives** | 4 | Provide an explicit statement of questions being addressed with reference to participants, interventions, comparisons, outcomes, and study design (PICOS). | Describe how disadvantage was defined if used as criterion in the review (e.g. for selecting studies, conducting analyses or judging applicability). | 3 |
|  | 4A |  | State the research questions being addressed with reference to health equity | 3 |
| **Methods** |  |  |  |  |
| **Protocol and registration** | 5 | Indicate if a review protocol exists, if and where it can be accessed (e.g., Web address), and, if available, provide registration information including registration number. |  | 3 |
| **Eligibility criteria** | 6 | 6. Specify study characteristics (e.g., PICOS, length of follow-up) and report characteristics (e.g., years considered, language, publication status) used as criteria for eligibility, giving rationale. | Describe the rationale for including particular study designs related to equity research questions. | 3 |
|  | 6A |  | Describe the rationale for including the outcomes - e.g. how these are relevant to reducing inequity. | 4 |
| **Information sources** | 7 | Describe all information sources (e.g., databases with dates of coverage, contact with study authors to identify additional studies) in the search and date last searched. | Describe information sources (e.g. health, non-health, and grey literature sources) that were searched that are of specific relevance to address the equity questions of the review. | 3 |
| **Search** | 8 | Present full electronic search strategy for at least one database, including any limits used, such that it could be repeated. | Describe the broad search strategy and terms used to address equity questions of the review. | 3  SM Tables 2-7 |
| **Study selection** | 9 | State the process for selecting studies (i.e., screening, eligibility, included in systematic review, and, if applicable, included in the meta-analysis). |  | 3-4 |
| **Data collection process** | 10 | Describe method of data extraction from reports (e.g., piloted forms, independently, in duplicate) and any processes for obtaining and confirming data from investigators. |  | 4  SM Table 8 |
| **Data items** | 11 | List and define all variables for which data were sought (e.g., PICOS, funding sources) and any assumptions and simplifications made. | List and define data items related to equity,where such data were sought (e.g. using PROGRESS-Plus or other criteria, context). | 5 |
| **Risk of bias in individual studies** | 12 | Describe methods used for assessing risk of bias of individual studies (including specification of whether this was done at the study or outcome level), and how this information is to be used in any data synthesis. |  | 4-5  SM Tables 9-10 |
| **Summary measures** | 13 | State the principal summary measures (e.g., risk ratio, difference in means). |  | 5 |
| **Synthesis of results** | 14 | Describe the methods of handling data and combining results of studies, if done, including measures of consistency (e.g., I^2^) for each meta-analysis. | Describe methods of synthesizing findings on health inequities (e.g. presenting both relative and absolute differences between groups). | 5 |
| **Risk of bias across studies** | 15 | 15. Specify any assessment of risk of bias that may affect the cumulative evidence (e.g., publication bias, selective reporting within studies). |  | 3-4 |
| **Additional analyses** | 16 | Describe methods of additional analyses (e.g., sensitivity or subgroup analyses, meta-regression), if done, indicating which were pre-specified. | Describe methods of additional synthesis approaches related to equity questions, if done, indicating which were pre-specified | 5 |
| **Results** |  |  |  |  |
| **Study selection** | 17 | Give numbers of studies screened, assessed for eligibility, and included in the review, with reasons for exclusions at each stage, ideally with a flow diagram. |  | 6 and Figure 1 |
| **Study characteristics** | 18 | For each study, present characteristics for which data were extracted (e.g., study size, PICOS, follow-up period) and provide the citations. | Present the population characteristics that relate to the equity questions across the relevant PROGRESS-Plus or other factors of interest. | Table 1 |
| **Risk of bias within studies** | 19 | Present data on risk of bias of each study and, if available, any outcome level assessment (see item 12). |  | Table 1 |
| **Results of individual studies** | 20 | For all outcomes considered (benefits or harms), present, for each study: (a) simple summary data for each intervention group (b) effect estimates and confidence intervals, ideally with a forest plot. |  | 6-10 |
| **Synthesis of results** | 21 | Present results of each meta-analysis done, including confidence intervals and measures of consistency. | Present the results of synthesizing findings on inequities (see 14). | Figures 3 and 4  SM Tables 11-17 |
| **Risk of bias across studies** | 22 | Present results of any assessment of risk of bias across studies (see Item 15). |  | Figure 2 |
| **Additional analysis** | 23 | Give results of additional analyses, if done (e.g., sensitivity or subgroup analyses, meta-regression [see Item 16]). | Give the results of additional synthesis approaches related to equity objectives, if done, (see 16). | Figures 3 and 4 |
| **Discussion** |  |  |  |  |
| **Summary of evidence** | 24 | Summarize the main findings including the strength of evidence for each main outcome; consider their relevance to key groups (e.g., healthcare providers, users, and policy makers). |  | 10-15 |
| **Limitations** | 25 | Discuss limitations at study and outcome level (e.g., risk of bias), and at review-level (e.g., incomplete retrieval of identified research, reporting bias). |  | 14-15 |
| **Conclusions** | 26 | Provide a general interpretation of the results in the context of other evidence, and implications for future research. | Present extent and limits of applicability to disadvantaged populations of interest and describe the evidence and logic underlying those judgments. | 15 |
|  | 26A |  | Provide implications for research, practice or policy related to equity where relevant (e.g. types of research needed to address unanswered questions). | 15 |
| **Funding** |  |  |  |  |
| **Funding** | 27 | Describe sources of funding for the systematic review and other support (e.g., supply of data); role of funders for the systematic review. |  | Title Page |

*From:* Source: Welch V, Petticrew M, Tugwell P, Moher D, O'Neill J, Waters E, White H, and the PRISMA-Equity Bellagio Group. (2012) [PRISMA-Equity 2012 Extension: Reporting Guidelines for Systematic Reviews with a Focus on Health Equity](http://www.plosmedicine.org/article/info%3Adoi%2F10.1371%2Fjournal.pmed.1001333). PLoS Med 9(10): e1001333. doi:10.1371/journal.pmed.1001333

For more information: <http://equity.cochrane.org/equity-extension-prisma>

### *Table 2. Search terms and strategy – MEDLINE (via PubMed website)*

|  | **Terms** |
| --- | --- |
| Exposure 1  **Physical Activity** | ("Physical activity" [tiab]) OR (Exercis* [tiab]) OR (Commut* [tiab]) OR ("activ* fisic*" [tiab] OR traslad* [tiab] OR ejercicio*[tiab]) OR (Physical activity [MeSH Terms]) OR (Exercise [MeSH Terms]) OR (Commuting [MeSH Terms]) |
| Exposure 2  **Diet** | (Nutrition* [tiab]) OR (Food* [tiab]) OR (Diet* [tiab]) OR (Eat*[tiab]) OR (Feed*[tiab]) OR "dietary intake*" [tiab] OR "energ* intake*" [tiab] OR "caloric intake*" [tiab] OR "nutriti* intake*" [tiab] OR "nutriti* assessment*" [tiab] OR "nutriti* survey*" [tiab] OR "nutriti* index*" [tiab] OR "nutriti* indices" [tiab] OR "nutriti* value*" [tiab] OR "nutriti* qualit*" [tiab] OR "dietary pattern*" [tiab] OR "dietary habit*") OR (alimento* [tiab] OR comer* [tiab] OR comida* [tiab] OR ingesta* [tiab] OR "habit* aliment*"[tiab]) OR ("Diet, Food, and Nutrition" [MeSH Terms]) OR (“Nutritional Sciences” [MeSH Terms]) OR ("Food" [MeSH Terms]) OR (“Diet" [MeSH Terms]) OR ("Eating" [MeSH Terms]) OR ("Feeding" [MeSH Terms]) |
| Exposure 3  **Sedentarism** | ((Sedentar* [tiab]) OR (("television watch*"[tiab]) OR ("TV watch*" [tiab]) OR ("screen watch*" [tiab]) OR ("computer" [tiab]) OR ("video* game*" [tiab])) OR (("sitting time"[tiab]) OR ("resting time" [tiab])) OR ("physical inactivity" [tiab]) OR (("ver television" OR "viendo TV" OR "frente a la pantalla*" OR "frente al computador*" OR "video* juego*" OR "sentad*" OR "descans*" OR "inactividad* fisica*" OR (Sedentary Lifestyle [MeSH Terms])) |
| Outcome  **Socioeconomic factors** | (Socioeconom* [tiab]) OR (“socioeconomic factors” [tiab]) OR (“socioeconomic status” [tiab]) OR (“socioeconomic position” [tiab]) OR (“social class*” [tiab]) OR (education* [tiab]) OR (occupation* [tiab]) OR (income* [tiab]) OR (employment* [tiab]) OR (poverty [tiab]) OR (poor* [tiab]) OR (deprivation* [tiab]) OR (deprived [tiab]) OR ("factor* socioeconomic*" OR "posicion* socioeconomic*" OR "clase* social*" OR educacion* OR ocupacion* OR ingreso* OR trabajo* OR empleo* OR pobre* OR deprivacion* OR deprivado* OR ("Socioeconomic factors" [MeSH Terms]) OR ("Educational status" [MeSH Terms]) OR ("Occupations" [MeSH Terms]) OR ("Income" [MeSH Terms]) OR ("Employment" [MeSH Terms]) OR ("Poverty" [MeSH Terms]) |
| Population  **Chilean** | ((Chile* [tiab] OR Chilean [tiab] OR Latinamerica* [tiab] OR “latin* americ*” [tiab] OR “South America” [tiab] OR “Southern Cone” [tiab] OR “America* del Sur” [tiab] OR “Sud America” [tiab] OR “Cono Sur” [tiab] OR Developing [tiab) AND Chile) OR (Chile [MeSH Terms]) |
| Search Strategy | [Exposure 1 OR Exposure 2 OR Exposure 3] AND Outcome AND Population |

### *Table 3. Search terms - Scopus*

| **Terms** |
| --- |
| ( TITLE-ABS-KEY ( "Physical* activ*" OR commut* OR exercis* ) ) OR ( TITLE-ABS-KEY ( "activ* fisic*" OR traslad* OR ejercicio* ) ) |
| ( TITLE-ABS-KEY ( nutrition* OR food* OR diet* OR eat* OR feed* OR "dietary intake*" OR "energ* intake*" OR "caloric intake*" OR "nutriti* intake*" OR "nutriti* assessment*" OR "nutriti* survey*" OR "nutriti* index*" OR "nutriti* indices" OR "nutriti* value*" OR "nutriti* qualit*" OR "dietary pattern*" OR "dietary habit*" ) ) OR ( TITLE-ABS-KEY (alimento* OR comer* OR comida* OR ingesta* OR "habit* aliment*" )) |
| ( TITLE-ABS-KEY ( sedentar* OR "television watch*" OR "TV watch*" OR "screen watch*" OR "computer" OR "video* game*" OR "sitting time" OR "resting time" OR "physical inactivity" ) ) OR ( TITLE-ABS-KEY ("ver W/3 television" OR "viendo W/3 TV" OR "frente W/3 pantalla*" OR "frente W/3 computador*" OR "video* juego*" OR "sentado*" OR "descans*" OR "inactividad* fisica*" ) ) |
| ( TITLE-ABS-KEY ( socioeconom* OR "socioeconomic factors" OR "socioeconomic status" OR "socioeconomic position" OR "social class*" OR education* OR occupation* OR income* OR employment* OR poverty OR poor* OR deprivation* OR deprived ) ) OR ( TITLE-ABS-KEY ( "factor* W/2 socioeconomic*" OR "posicion* W/2 socioeconomic*" OR "clase* social*" OR educacion* OR ocupacion* OR ingreso* OR trabajo* OR empleo* OR pobre* OR deprivacion* OR deprivado* ) ) |
| ( TITLE-ABS-KEY ( chile* OR chilean OR latinamerica* OR "latin* americ*" OR latinoamerica OR "South America" OR “Southern Cone” OR "America* del Sur" OR "Sud America" OR “Cono Sur” OR developing ) ) AND ( ALL ( chile ) ) |

### *Table 4. Search terms - Web of Science*

| **Terms** |
| --- |
| TS= ("Physical* activ*" OR commut* OR exercis*) OR TS= ("activ* fisic*" OR traslad* OR ejercicio*) |
| TS= (nutrition* OR food* OR diet* OR eat* OR feed* OR "dietary intake*" OR "energ* intake*" OR "caloric intake*" OR "nutriti* intake*" OR "nutriti* assessment*" OR "nutriti* survey*" OR "nutriti* index*" OR "nutriti* indices" OR "nutriti* value*" OR "nutriti* qualit*" OR "dietary pattern*" OR "dietary habit*") |
| TS= (sedentar* OR "television watch*” OR "TV watch*” OR "screen watch*" OR "computer" OR "video* game*" OR "sitting time" OR "resting time" OR "physical inactivity") OR TS= ("ver NEAR/3 television" OR "viendo NEAR /3 TV" OR "frente NEAR /3 pantalla*" OR "frente NEAR/3 computador*" OR "video* juego*" OR "sentado*" OR "descans*" OR "inactividad* fisica*" ) |
| TS= (socioeconom* OR "socioeconomic factors" OR "socioeconomic status" OR "socioeconomic position" OR "social class*" OR education* OR occupation* OR income* OR employment* OR poverty OR poor* OR deprivation* OR deprived) OR TS= ( "factor* NEAR/2 socioeconomic*" OR "posicion* NEAR /2 socioeconomic*" OR "clase* social*" OR educacion* OR ocupacion* OR ingreso* OR trabajo* OR empleo* OR pobre* OR deprivacion* OR deprivado* ) |
| TS= (Chile* OR chilean OR latinamerica* OR "latin* America*" OR "South America" OR "America* del Sur" OR "Sud America" OR “Cono Sur” OR “Southern Cone” OR developing) AND (CU= (Chile) OR TS=(Chile))) |

### *Table 5. Search terms - PsyINFO (via Ovid)*

| **Terms** |
| --- |
| (physical activity or "Exercis*" or "Commut*" or "activ* fisic*" or "traslad*" or "ejercicio*").ab,ti. or (Physical activity or Exercise or Commuting).mh,sh. |
| ("Nutrition*" or "Food*" or "Diet*" or "Eat*" or "Feed*" or "dietary intake*" or "energ* intake*" or "caloric intake*" or "nutriti* intake*" or "nutriti* assessment*" or "nutriti* survey*" or "nutriti* index*" or "nutriti* indices" or "nutriti* value*" or "nutriti* qualit*" or "dietary pattern*" or "dietary habit*" or ("alimento*" or "comer*" or "comida*" or "ingesta*" or "habit* aliment*")).ab,ti. or (Diet or Nutrition or Food or "Eating Behavior" or "Food Intake" or "Eating Attitudes").mh,sh. |
| ("Sedentar*" or "television watch*" or "TV watch*" or "screen watch*" or "computer" or "video* game*" or "sitting time" or "resting time" or "physical inactivity" or ("ver ADJ3 television" or "viendo ADJ3 TV" or "frente ADJ3 pantalla*" or "frente ADJ3 computador*" or "video* juego*" or "sentad*" or "descans*" or "inactividad* fisica*")).ab,ti. or Sedentary behaviour.mh,sh. |
| ("Socioeconom*" or "socioeconomic factors" or "socioeconomic status" or "socioeconomic position" or "social class*" or "education*" or "occupation*" or "income*" or "employment*" or poverty or "poor*" or "deprivation*" or deprived or "factor* ADJ2 socioeconomic*" or "posicion* ADJ2 socioeconomic*" or "clase* social*" or "educacion*" or "ocupacion*" or "ingreso*" or "trabajo*" or "empleo*" or "pobre*" or "deprivacion*" or "deprivado*").ab,ti. or (Socioeconomic level or socioeconomic class or socioeconomic status or Educational Attainment Level or Occupations or Income level or Employment status or Poverty).mh,sh. |
| ("Chile*" or "Chilean" or "Latinamerica*" or "latin* americ*" or "South America" or "Southern Cone" or "America* del Sur" or "Sud America" or "Cono Sur" or Developing).ab,ti. and chile.af. |

### *Table 6. Search terms - LILACS (via BVS)*

| **Terms** |
| --- |
| (tw:(“Physical activity” OR commut$ OR exercis$ OR "actividad fisica" OR traslad$ OR ejercicio$)) |
| (tw:(Nutrition$ OR Food$ OR Diet$ OR Eat$ OR Feed$ OR "dietary intake" OR "energy intake" OR "caloric intake" OR "nutritional intake" OR "nutritional assessment" OR " nutritional survey" OR " nutritional index" OR "nutritional indices" OR "nutritional value" OR "nutritional quality" OR "dietary pattern" OR "dietary habit" OR alimento$ OR comer$ OR comida$ OR ingesta$ OR "habito alimentario" OR "habito alimenticio")) |
| (tw:(Sedentar$ OR "television watch" OR "TV watch" OR "screen watch" OR computer$ OR "video game" OR "sitting time" OR "resting time" OR "physical inactivity" OR "ver television" OR "viendo TV" OR "frente pantalla" OR "frente computador" OR "video juego" OR sentad$ OR descans$ OR "inactividad fisica")) |
| (tw:(Socioeconom$ OR "socioeconomic factors" OR "socioeconomic status" OR "socioeconomic position" OR "social class" OR education$ OR occupation$ OR income$ OR employment$ OR poverty OR poor$ OR deprivation$ OR deprived OR "factor socioeconomico" OR "posicion socioeconomica" OR "clase social" OR educacion$ OR ocupacion$ OR ingreso$ OR trabajo$ OR empleo$ OR pobre$ OR deprivacion$ OR deprivado$)) |
| tw:(Chile$ OR Chilean OR Latinamerica$ OR "latin america" OR "South America" OR "Southern Cone" OR "America del Sur" OR "Sud America" OR "Cono Sur" OR Developing) AND ((cp:(chile)) OR (pais_afiliacao:(chile)) OR (pais_assunto:(chile))) |

### *Table 7. Search terms and strategy – Grey literature*

|  | **Terms** |
| --- | --- |
|  |  |
| Exposure 1  **Physical Activity** | ("Physical activity" OR Exercise OR Commuting OR "actividad fisica” OR OR ejercicio) |
| Exposure 2  **Diet** | (Nutrition OR Food OR Diet OR Eat OR Feeding OR "dietary intake" OR "energy intake" OR "caloric intake" OR "dietary pattern" OR "dietary habit") OR (alimentación OR comer OR comida OR ingesta OR "hábito alimentario" |
| Exposure 3  **Sedentarism** | (Sedentary OR "television watching OR "TV watching" OR "screen watching" OR "computer" OR "video game" OR "sitting time" OR "resting time" OR "physical inactivity" OR "ver television" OR "viendo TV" OR "frente a la pantalla" OR "frente al computador" OR "video juego" OR "sentado" OR "descanso" OR "inactividad fisica" |
| Outcome  **Socioeconomic factors** | (Socioeconomic OR "socioeconomic factors" OR "socioeconomic status" OR "socioeconomic position" OR "social class" OR education OR occupation OR income OR employment OR poverty OR poor OR deprivation OR deprived OR "factor socioeconomico" OR "posición socioeconomica" OR "clase social" OR educacion OR ocupacion OR ingreso OR trabajo OR empleo OR pobre OR deprivacion OR deprivado) |
| Population  **Chilean** | Chile OR Chilean OR Latinamerica OR "latin america" OR "South America" OR "Southern Cone" OR "America del Sur" OR "Sud America" OR "Cono Sur" OR Developing |
| General search strategy | (diet OR physical activity OR sedentarism AND socioeconomic position AND Chile) |
| Specific search Strategy | [Exposure 1 OR Exposure 2 OR Exposure 3] AND Outcome AND Population |

### *Table 8. Data extraction fields*

- Author
- Journal
- Year of publication
- Location of study (country/region/city)
- Study design
- Study/survey name
- Date of data collection
- Sample population (children/adolescents/adults)
- Sampling recruitment techniques
- Sample size
- Study response rate
- Population and participant characteristics
- Sample’s age
- SEP indicator
- SEP assessment method
- Dietary factors
- Dietary assessment method
- Direction and magnitude of the association between SEP and diet

### *Table 9. Newcastle - Ottawa Quality Assessment Scale (adapted for cross sectional studies) (10 points max.)*

**Selection:** (Maximum 5 stars)

1. Representativeness of the sample:
2. Truly representative of the average in the target population* (1 star)

- Clear sample frame
- Multistage sampling
- Random sampling in all sampling stages

1. Somewhat representative of the average in the target population* (0.5 star)

- Single-stage sampling
- Non-random sampling on at least 1 sampling stages (e.g. random of students with a school but non-random sampling of schools)

1. Selected group of participants (0 star)

- Convenience sampling or selection based on a particular characteristic of individuals

1. No description of the sampling strategy (0 star)
2. Sample size:
3. Justified and satisfactory * (1 star)
   - Sample size justified and estimated with effect design formula
4. Justified but not satisfactory (0.5 star)
   - Justified but not estimated with effect design formula
5. Not justified (0 star)
6. Non-respondents:
7. Comparability between respondents and non-respondents’ characteristics is established, and the response rate is satisfactory* (1 star)

- Non-response bias measures (0.5 star)
- ≥85% Response rate (0.5 star)

1. The response rate is unsatisfactory, or the comparability between respondents and non-respondents is unsatisfactory (0 star)

- No description of non-respondents or measures for non-response bias
- <85% Response rate

1. No description of the response rate or the characteristics of the responders and the non-responders (0 star)
2. Ascertainment of the exposure (risk factor):
3. Validated measurement tool** (2 stars)
4. Non-validated measurement tool, but the tool is available or described* (1 star)
5. No description of the measurement tool (0 star)

**Comparability:** (Maximum 2 stars)

1. The subjects in different outcome groups are comparable, based on the study design or analysis.
2. The study compares the outcome groups by at least two socioeconomic groups (select one outcome to compare)* (1 star)
3. The study controls the bivariate association of outcome and exposure for any additional factor (gender, body weight)* (1 star)

**Outcome:** (Maximum 3 stars)

1. Assessment of the outcome:
2. Record (accelerometer)** (2 stars)
3. Self-report diary (activities or food)** (2 stars)
4. Self-report recall (24-recall)* (1 star)
5. Self-report questionnaire (food-frequency questionnaire, physical activity questionnaire)* (1 star)
6. No description
7. Statistical test:
8. The statistical test used to analyse the data is clearly described and appropriate, and the measurement of the association is presented, including confidence intervals and the probability level (p value)* (1 star)
9. The statistical test is not appropriate, not described or incomplete.

### *Table 10. NOQAS quality assessment per component of each included publications*

| **Author** | **Year data collection** | **Representativeness of the sample** | **Sample size** | **Non-respondents** | **Assessment of the exposure** | **Comparability** | **Assessment of the outcome** | **Statistical test** | **Total quality score** |
| --- | --- | --- | --- | --- | --- | --- | --- | --- | --- |
| Adjemian et al., (2007) | N/R | 0,5 | 0 | 0 | 1 | 1 | 1 | 0 | 3,5 |
| Correa-Burrows et al., (2015) | 2010 | 1 | 0,5 | 0 | 2 | 1 | 1 | 1 | 6,5 |
| Essman et al., (2018) / FEChiC and GOCS | 2016 | 0,5 | 0,5 | 1 | 2 | 1 | 1 | 1 | 6,5 |
| Hoffmeister et al., (2016) | 2009-2010 | 1 | 0,5 | 0 | 0 | 1 | 1 | 1 | 4,5 |
| Ivanovic et al., (1991) | 1982 | 0,5 | 0 | 0 | 2 | 1 | 1 | 1 | 5,5 |
| Ivanovic et al., (1992) | 1986-1987-1989 | 1 | 0 | 0 | 2 | 1 | 1 | 0 | 5 |
| Jensen et al., (2019) / FEChiC and GOCS | 2016 | 0,5 | 0,5 | 1 | 2 | 1 | 1 | 1 | 6,5 |
| Liberona (2011) | 2007 | 0 | 0,5 | 0,5 | 2 | 1 | 1 | 1 | 6 |
| Cediel et al., (2018) / ENCA 2010-2011 | 2010-2011 | 1 | 1 | 0,5 | 2 | 1 | 1 | 1 | 7,5 |
| Celis-Morales et al., (2011) | 2008 | 0 | 0 | 0 | 1 | 1 | 2 | 1 | 5 |
| Duran-Aguero et al., (2015) | 2014 | 0 | 0,5 | 0 | 2 | 1 | 1 | 0 | 4,5 |
| Echeverria et al., (2016) | 2010-2014 | 0 | 0 | 0 | 1 | 0 | 1 | 0 | 2 |
| Fisberg et al., (2018) | 2014-2015 | 1 | 1 | 0 | 2 | 1 | 1 | 0 | 6 |
| Gomez et al., (2019) / ELANS | 2014-2015 | 1 | 1 | 0 | 2 | 1 | 1 | 1 | 6 |
| Chilean National Quality of Life and Health Survey 2006 | 2006 | 1 | 0,5 | 0,5 | 2 | 1 | 1 | 1 | 7 |
| Chilean National Quality of Life and Health Workers Survey 2009-2010 | 2009-2010 | 1 | 0,5 | 0,5 | 2 | 1 | 1 | 1 | 7 |
| Chilean National Health Survey 2009-2010 | 2009-2010 | 1 | 1 | 1 | 2 | 2 | 1 | 1 | 9 |
| Chilean National Food Intake Survey 2014 | 2010-2011 | 1 | 1 | 0,5 | 2 | 2 | 1 | 1 | 8,5 |
| Chilean National Health Survey 2016-2017 | 2016-2017 | 1 | 1 | 0 | 2 | 1 | 1 | 0 | 6 |
| Pinto et al., (2019) / ELANS | 2014-2015 | 1 | 1 | 0 | 2 | 1 | 1 | 0 | 5 |
| Ratner et al., (2008) | N/R | 0 | 0 | 0 | 2 | 1 | 1 | 0 | 4 |

NOQAS: Newcastle Ottawa Quality Assessment Scale

### *Table 11. Summary of relative differences and/or odds ratios from articles assessing associations between energy intake, protein and carbohydrate intake and socioeconomic position indicators+*

| **Study name / Author** | **Sample population** | **Dietary indicator** | **Education** | **Income** | **Index** |
| --- | --- | --- | --- | --- | --- |
| ***Energy intake*** | | | | | |
| Adjemian et al., (2007) | Children | Kcal/d |  |  | -4.8% |
| Essman et al., (2018) / FEChiC | Children | Kcal/d | -0.3% |  |  |
| Essman et al., (2018) / GOCS | Children | Kcal/d | -2.0% |  |  |
| Ivanovic et al., (1991) | Children | % adequacy nutrient intake |  |  | -17.2%* |
| Liberona (2011) | Children | Kcal/d |  |  | -0.8% |
| ENCA 2010 | Adults | [Men] Kcal/d |  |  | +8.6% |
| ENCA 2010 | Adults | [Women] Kcal/d |  |  | -4.3% |
| Celis-Morales et al., (2011) / GENADIO | Adults | Kcal/d | +22.1%* |  | +20.6%* |
| ***Protein intake*** | | | | | |
| Adjemian et al., (2007) | Children | g/d |  |  | -1.7% |
| Ivanovic et al., (1991) | Children | % adequacy nutrient intake |  |  | -37.7%* |
| Ivanovic et al., (1992) | Children | % adequacy nutrient intake |  |  | -13.0%* |
| Ivanovic et al., (1992) | Children | % of total EI |  |  | -9.7% |
| Liberona (2011) | Children | g/d |  |  | -15.0%* |
| ENCA 2010 | Adults | [Men] g/d |  |  | -7.3% |
| ENCA 2010 | Adults | [Women] g/d |  |  | -10.4%* |
| Celis-Morales et al., (2011) / GENADIO | Adults | g/d | +5.4% |  | +9.0% |
| ***Carbohydrate intake*** | | | | | |
| Adjemian et al., (2007) | Children | g/d CHO |  |  | -3.5% |
| Ivanovic et al., (1991) | Children | % of total EI |  |  | +17.3%* |
| Ivanovic et al., (1992) | Children | % of total EI |  |  | -3.7% |
| Liberona (2011) | Children | g/d CHO |  |  | +6.8% |
| ENCA 2010 | Adults | [Men] g/d CHO |  |  | +7.6% |
| ENCA 2010 | Adults | [Women] g/d CHO |  |  | -1.3% |
| Celis-Morales et al., (2011) / GENADIO | Adults | g/d CHO | +16.8%* |  | +18.4%* |

+Relative differences (%) in intakes between lowest and highest socioeconomic groups were estimated by: ([value highest SEP group – value lowest SEP group] / value highest SEP group) X 100. Odds ratio (OR) were reported for dichotomous outcome variables. Reference category for OR: Highest SEP group. *Relative differences >10% in intakes, or p<0.05 for OR.

Differences reported are for the lowest socioeconomic group relative to the highest group (i.e.: ‘-’ refers to lower intakes among the lower SEP group relative to the highest SEP group (or higher intakes among the highest SEP group relative to the lowest SEP group); ‘+‘ refers to higher intakes among the lower SEP group relative to the lowest SEP group (or lower intakes among the highest SEP group relative to the lowest SEP group).

Kcal: kilocalorie; d: day; g: grams; EI: energy intake. % Adequacy nutrient intake: % compliance with the energy and protein intake recommendations by age and gender defined by FAO/WHO/UNU (1985)^57^.

### *Table 12. Summary of relative differences and/or odds ratios from articles assessing associations between total fat and saturated fat, fibre and sugar intake and socioeconomic position+*

| **Study name / Author** | **Sample population** | **Dietary indicator** | **Education** | **Income** | **Index** |
| --- | --- | --- | --- | --- | --- |
| ***Total fat*** | | | | | |
| Adjemian et al., (2007) | Children | g/d total fat |  |  | -10.9%* |
| Ivanovic et al., (1991) | Children | % of total EI |  |  | -25.5%* |
| Ivanovic et al., (1992) | Children | % of total EI |  |  | -13.3%* |
| Liberona (2011) | Children | g/d total fat |  |  | -8.6% |
| ENCA 2010 | Adults | [Men] g/d total fat |  |  | +2.0% |
| ENCA 2010 | Adults | [Women] g/d total fat |  |  | -3.8% |
| Celis-Morales et al., (2011) / GENADIO | Adults | g/d total fat | +37.2%* |  | +29.0%* |
|  |  | ***Saturated fat*** |  |  |  |
| Liberona (2011) | Children | g/d saturated fat |  |  | -8.6% |
| ENCA 2010 | Adults | [Men] g/d  saturated fat |  |  | -2.4% |
| ENCA 2010 | Adults | [Women] g/d saturated fat |  |  | -16.3%* |
| ***Fibre intake*** | | | | | |
| Liberona (2011) | Children | g/d fibre |  |  | +3.5% |
| ENCA 2010 | Adults | [Men]  g/d fibre |  |  | -12.0%* |
| ENCA 2010 | Adults | [Women]  g/d fibre |  |  | -15.7%* |
| Celis-Morales et al., (2011) / GENADIO | Adults | g/d fibre | +59.6%* |  | +44.7%* |
| ***Sugar*** | | | | | |
| Liberona (2011) | Children | g/d sugar |  |  | +35.2%* |
| ENCA 2010 | Adults | g/d sugar |  |  | -16.5%* |
| Fisberg et al., (2018) / ELANS | Adults | g/d sugar |  |  | +11.4%* |
| Fisberg et al., (2018) / ELANS | Adults | % of total EI |  |  | +1.5% |
| Fisberg et al., (2018) / ELANS | Adults | % of total CHO |  |  | -2.7% |

+Relative differences (%) in intakes between lowest and highest socioeconomic groups were estimated by: ([value highest SEP group – value lowest SEP group] / value highest SEP group) X 100. Odds ratio (OR) were reported for dichotomous outcome variables. Reference category for OR: Highest SEP group. *Relative differences >10% in intakes, or p<0.05 for OR.

Differences reported are for the lowest socioeconomic group relative to the highest group (i.e.: ‘-’ refers to lower intakes among the lower SEP group relative to the highest SEP group (or higher intakes among the highest SEP group relative to the lowest SEP group); ‘+‘ refers to higher intakes among the lower SEP group relative to the lowest SEP group (or lower intakes among the highest SEP group relative to the lowest SEP group).

d: per day; g: grams; EI: energy intake. % of total energy intake (EI): % from macronutrient contributing to 100% of EI. % of total CHO: % from sugar contributing to 100% of CHO.

### *Table 13. Summary of relative differences and/or odds ratios from articles assessing associations between fruits, vegetables, and fruits and vegetables (FVs) consumption and socioeconomic position+*

| **Study name / Author** | **Sample population** | **Dietary indicator** | **Education** | **Income** | **Index** |
| --- | --- | --- | --- | --- | --- |
| ***Fruits*** | | | | | |
| Liberona (2011) | Children | g fruits/d |  |  | -23.9%* |
| ENCA 2010 | Adults | g fruits/d |  |  | -31.1%* |
| ENCA 2010 | Adults | % ever fruits consumption/m |  |  | -OR 0.22* |
| ENCAVI 2006 | Adults | % fruits consumption everyday |  | -OR 0.57* |  |
| ENS 2009-2010 | Adults | % fruits consumption everyday | -OR 0.92 |  |  |
| Ratner et al., (2008) | Adults | % ≥2 times/d fruits | +OR 1.42 |  |  |
| ***Vegetables*** | | | | | |
| Liberona (2011) | Children | g vegetables/d |  |  | -31.8%* |
| ENCA 2010 | Adults | g vegs/d |  |  | -20.5%* |
| ENCA 2010 | Adults | % ever vegs consumption/m |  |  | OR 1.00 |
| ENCAVI 2006 | Adults | % vegs consumption everyday |  | -OR 0.32* |  |
| ENS 2009-2010 | Adults | % vegs consumption everyday | -OR 0.81 |  |  |
| Ratner et al., (2008) | Adults | % ≥2 times/d vegs | +OR 1.24 |  |  |
| ***Fruits and Vegetables*** | | | | | |
| ENCA 2010 | Adults | % ≥5 portions/d FVs |  |  | -OR 0.47* |
| ENS 2009-2010 | Adults | % ≥5 portions/d FVs | -OR 0.81 |  |  |
| ENS 2009-2010 | Adults | [Men] % ≥5 portions/d FVs | -OR 0.84 |  |  |
| ENS 2009-2010 | Adults | [Women] % ≥5 portions/d FVs | -OR 0.73 |  |  |
| ENS 2016-2017 | Adults | % ≥5 portions of fruit or vegetable daily | -OR 0.65 |  |  |
| ENS 2009-2010 | Adults | g FVs/d | -9.1% |  |  |
| ENS 2009-2010 | Adults | [Men] g FVs/d | -0.8% |  |  |
| ENS 2009-2010 | Adults | [Women] g FVs/d | -16.8%* |  |  |

+Relative differences (%) in intakes between lowest and highest socioeconomic groups were estimated by: ([value highest SEP group – value lowest SEP group] / value highest SEP group) X 100. Odds ratio (OR) were reported for dichotomous outcome variables. Reference category for OR: Highest SEP group.*Relative differences >10% in intakes, or p<0.05 for OR.

Differences reported are for the lowest socioeconomic group relative to the highest group (i.e.: ‘-’ refers to lower intakes among the lower SEP group relative to the highest SEP group (or higher intakes among the highest SEP group relative to the lowest SEP group); ‘+‘ refers to higher intakes among the lower SEP group relative to the lowest SEP group (or lower intakes among the highest SEP group relative to the lowest SEP group).

d: per day; m: per month; g: grams; EI: energy intake.

### *Table 14. Summary of relative differences and/or odds ratios from articles assessing associations between dairy products, pulses, fish/seafood consumption and socioeconomic position+*

| **Study name / Author** | **Sample population** | **Dietary indicator** | **Education** | **Income** | **Index** |
| --- | --- | --- | --- | --- | --- |
| ***Dairy products*** | | | | | |
| Liberona (2011) | Children | g dairy/d |  |  | -32.4%* |
| ENCA 2010 | Adults | % ≥3 portions/d dairy |  |  | -OR 0.42* |
| ENCAVI 2006 | Adults | % dairy consumption everyday |  | -OR 0.22* |  |
| ENS 2009-2010 | Adults | % ≥3 portions of dairy daily | -OR 0.58 |  |  |
| ENS 2016-2017 | Adults | % ever low-fat dairy consumption | -OR 0.46* |  |  |
| ENS 2016-2017 | Adults | % ≥3 portions/d low-fat dairy | -OR 0.41 |  |  |
| Ratner et al., (2008) | Adults | % ≥2 times/d dairy | -OR 0.88 |  |  |
| ***Pulses*** | | | | | |
| Liberona (2011) | Children | g pulses/d |  |  | +89.3%* |
| ENCA 2010 | Adults | % ever pulses consumption |  |  | -OR 0.92 |
| ENCA 2010 | Adults | g pulses/d |  |  | +16.4%* |
| ENCA 2010 | Adults | % ≥2 portions/wk pulses |  |  | +OR 1.53 |
| ENCAVI 2006 | Adults | % 2-3 days/wk pulses |  | +OR 2.38* |  |
| ENS 2016-2017 | Adults | % ≥2 portions/wk pulses | +OR 1.53 |  |  |
| Ratner et al., (2008) | Adults | % ≥2 times/wk pulses | +OR 1.72 |  |  |
| ***Fish / Seafood*** | | | | | |
| Liberona (2011) | Children | g fish/d |  |  | -45.5%* |
| ENCA 2010 | Adults | % ≥2 times/wk fish |  |  | -OR 0.32* |
| ENCAVI 2006 | Adults | % 2-3 days/wk fish |  | -OR 0.88 |  |
| ENS 2009-2010 | Adults | % ever Fish or seafood/wk | -OR 0.53* |  |  |
| ENS 2009-2010 | Adults | [Men] % ever Fish or seafood/wk | -OR 0.45* |  |  |
| ENS 2009-2010 | Adults | [Women] % ever Fish or seafood/wk | -OR 0.60 |  |  |
| ENS 2016-2017 | Adults | % ≥2 times/wk fish | -OR 0.52 |  |  |
| Ratner et al., (2008) | Adults | % ≥2 times/wk fish | -OR 0.83 |  |  |

+Relative differences (%) in intakes between lowest and highest socioeconomic groups were estimated by: ([value highest SEP group – value lowest SEP group] / value highest SEP group) X 100. Odds ratio (OR) were reported for dichotomous outcome variables. Reference category for OR: Highest SEP group.*Relative differences >10% in intakes, or p<0.05 for OR.

Differences reported are for the lowest socioeconomic group relative to the highest group (i.e.: ‘-’ refers to lower intakes among the lower SEP group relative to the highest SEP group (or higher intakes among the highest SEP group relative to the lowest SEP group); ‘+‘ refers to higher intakes among the lower SEP group relative to the lowest SEP group (or lower intakes among the highest SEP group relative to the lowest SEP group).

d: per day; wk: per week; g: grams; EI: energy intake.

### *Table 15. Summary of relative differences and/or odds ratios from articles assessing associations between wholegrains, ultra-processed food and fried food, and sugar-sweetened beverage consumption and socioeconomic position+*

| **Study name / Author** | **Sample population** | **Dietary indicator** | **Education** | **Income** | **Index** | |
| --- | --- | --- | --- | --- | --- | --- |
| ***Wholegrains*** | | | | | | |
| ENS 2009-2010 | Adults | % Wholegrains/d | -OR 0.26* |  |  | |
| ENS 2009-2010 | Adults | [Men] % Wholegrains/d | -OR 0.25* |  |  | |
| ENS 2009-2010 | Adults | [Women] % Wholegrains/d | -OR 0.27* |  |  | |
| ***Ultra-processed food / Fried food*** | | | | | |  |
| Cediel et.al (2018) / ENCA 2010 | Adults | % of total EI from ultra-processed food | 5.0% | +21.4%* |  | |
| ENCAVI 2006 | Adults | % 1 day/wk fried food |  | +OR 1.11 |  | |
| Ratner et al., (2008) | Adults | % 3-6 times/wk fried food | -OR 0.81 |  |  | |
| ***Sugar-sweetened beverages*** | | | | | | |
| Essman et al., (2018) / FEChiC | Children | Kcal/d from beverages | -6.4% |  |  | |
| Essman et al., (2018) / GOCS | Children | Kcal/d from beverages | +17.2%* |  |  | |
| Essman et al., (2018) / FEChiC | Children | % of total EI from high in SSB | +0.8% |  |  | |
| Essman et al., (2018) / GOCS | Children | % of total EI from high in SSB | +11.5%* |  |  | |
| Hoffmeister et al., (2016) | Children | [2 y-o] % always/d SSBs | +OR 2.71* |  |  | |
| Hoffmeister et al., (2016) | Children | [4 y-o] % always/d SSBs | +OR 1.45 |  |  | |
| Hoffmeister et al., (2016) | Children | [2 y-o] % always before bed/d SSBs | +OR 2.00* |  |  | |
| Hoffmeister et al., (2016) | Children | [4 y-o] % always before bed/d SSBs | +OR 2.74* |  |  | |
| Liberona (2011) | Children | ml/d SSBs |  |  | -3.0% | |
| ENCA 2010 | Adults | % ever SSBs consumption |  |  | +OR 1.61 | |
| ENCA 2010 | Adults | ml/d SSBs |  |  | -32.9%* | |
| ENCA 2010 | Adults | g sugar/day from SSBs |  |  | -28.8%* | |
| ENCAVI 2006 | Adults | % fizzy drinks/everyday |  | -OR 0.62 |  | |
| ENS 2016-2017 | Adults | % ≥1 glass/d SSBs | -OR 0.96 |  |  | |

+Relative differences (%) in intakes between lowest and highest socioeconomic groups were estimated by: ([value highest SEP group – value lowest SEP group] / value highest SEP group) X 100. Odds ratio (OR) were reported for dichotomous outcome variables. Reference category for OR: Highest SEP group.*Relative differences >10% in intakes, or p<0.05 for OR.

Differences reported are for the lowest socioeconomic group relative to the highest group (i.e.: ‘-’ refers to lower intakes among the lower SEP group relative to the highest SEP group (or higher intakes among the highest SEP group relative to the lowest SEP group); ‘+‘ refers to higher intakes among the lower SEP group relative to the lowest SEP group (or lower intakes among the highest SEP group relative to the lowest SEP group).

d: per day; wk: per week; g: grams; ml: millilitres; EI: energy intake. % of total energy intake (EI): % from food group contributing to 100% of EI. High in SSB: Beverage high in sugar, calories, fat and/or sodium according to 2016 Chilean food and drink regulation.

### *Table 16. Summary of relative differences and/or odds ratios from articles assessing associations between non-nutritive sweetener consumption, dietary patterns and socioeconomic position+*

| **Study name / Author** | **Sample population** | **Dietary indicator** | **Education** | | **Income** | | **Index** |  |
| --- | --- | --- | --- | --- | --- | --- | --- | --- |
| ***Non-nutritive sweeteners*** | | | | | | | |  |
| Essman et al., (2018) / FEChiC | Children | % of total EI from low in SSB | -0.8% |  | |  | | |
| Essman et al., (2018) / GOCS | Children | % of total EI from low in SSB | -11.5%* |  | |  | | |
| ENCA 2010 | Adults | % ever non-caloric beverages consumption |  |  | | -OR 0.11* | | |
| ENCA 2010 | Adults | ml/d Non-caloric beverages |  |  | | -46.0%* | | |
| ENCA 2010 | Adults | % ever non-nutritive sweetener consumption |  |  | | -OR 0.28* | | |
| Duran-Aguero et al., (2015) | Adults | g/d stevia | +5.3% |  | |  | | |
| Duran-Aguero et al., (2015) | Adults | kg/d stevia | +0.0% |  | |  | | |
| ***Dietary patterns*** | | | | | | | | |
| Correa-Burrows et al., (2015) | Children | % Unhealthy snacking at school  (amounts of saturated fat, fibre, sugar and salt in the foods) |  |  | | +OR 1.38 | | |
| ENCA 2010 | Adults | % Unhealthy eating  (SHEI) |  |  | | +OR 1.51 | | |
| Echeverria et al., (2016) | Adults | % Lower adherence to Med diet | +OR 1.83* |  | |  | | |
| Echeverria et al., (2016) | Adults | Score adherence to Med diet | -10.9%* |  | |  | | |
| Gomez, G. et al., (2019) / ELANS | Adults | Score higher consumption of 10 healthy dietary items↑ |  |  | | -12.6%* | | |
| Gomez, G. et al., (2019) / ELANS | Adults | Score lower consumption of 7 unhealthy dietary items↓ |  |  | | +1.7% | | |
| Gomez, G. et al., (2019) / ELANS | Adults | Score 10 healthy and 7 unhealthy dietary items↑↓ |  |  | | -7.2% | | |
| Pinto, V. et al., (2019) / ELANS | Adults | % Low Healthy Eating Index (AHEI-2010) |  |  | | +OR 12.63* | | |
| Pinto, V. et al., (2019) / ELANS | Adults | % High Healthy Eating Index (AHEI-2010) |  |  | | +OR 1.70 | | |

+Relative differences (%) in intakes between lowest and highest socioeconomic groups were estimated by: ([value highest SEP group – value lowest SEP group] / value highest SEP group) X 100. Odds ratio (OR) were reported for dichotomous outcome variables. Reference category for OR: Highest SEP group.*Relative differences >10% in intakes, or p<0.05 for OR.

Differences reported are for the lowest socioeconomic group relative to the highest group (i.e.: ‘-’ refers to lower intakes among the lower SEP group relative to the highest SEP group (or higher intakes among the highest SEP group relative to the lowest SEP group); ‘+‘ refers to higher intakes among the lower SEP group relative to the lowest SEP group (or lower intakes among the highest SEP group relative to the lowest SEP group).

d: per day; wk: per week; g: grams; ml: millilitres; EI: energy intake. % of total energy intake (EI): % from food group contributing to 100% of EI. Low in SSB: Beverage low in sugar, calories, fat and/or sodium according to 2016 Chilean food and drink regulation; SHEI: Spanish Healthy Eating Index; AHEI: Alternate Healthy Eating Index; Med: Meditarranean

↑ fruits, vegetables, beans and legumes, nuts and seeds, whole grains, milk, total polyunsaturated fatty acids, fish, plant omega-3s, and dietary fiber

↓ unprocessed read meats, processed meats, sugar-sweetened beverages, saturated fat, trans fat, dietary cholesterol, and sodium

### *Table 17. Summary of relative differences and/or odds ratios from articles assessing associations between meal consumption and socioeconomic position+*

| **Study name / Author** | **Sample population** | **Dietary indicator** | **Education** | **Income** | **Index** | **Occupation** |  |
| --- | --- | --- | --- | --- | --- | --- | --- |
| ***Meal consumption*** | | | | | | | |
| Jensen et al., (2019) / FEChiC | Children | Snacks eating occasions/d | -1.7% |  |  |  |  |
| Jensen et al., (2019) / GOCS | Children | Snacks eating occasions/d | +2.3% |  |  |  |  |
| Jensen et al., (2019) / FEChiC | Children | Meals eating occasions/d | -2.4% |  |  |  |  |
| Jensen et al., (2019) / GOCS | Children | Meals eating occasions/d | -6.3% |  |  |  |  |
| ENCA 2010 | Adults | % Breakfast consumption previous day |  |  | +OR 1.13 |  |  |
| ENCA 2010 | Adults | % Lunch consumption  previous day |  |  | -OR 0.80 |  |  |
| ENCA 2010 | Adults | % Tea-time consumption previous day |  |  | +OR 1.27 |  |  |
| ENCA 2010 | Adults | % Dinner consumption previous day |  |  | -OR 0.76 |  |  |
| ENCAVI 2006 | Adults | % Breakfast consumption everyday |  | -OR 0.81 |  |  |  |
| ENETS 2009-2010 | Adults | % [Male] Breakfast consumption everyday | -OR 0.90 | -OR 0.40* |  | -OR 0.86 |  |
| ENETS 2009-2010 | Adults | % [Women] Breakfast consumption everyday | +OR 2.03 | -OR 0.51 |  | +OR 1.31 |  |

+Relative differences (%) in intakes between lowest and highest socioeconomic groups were estimated by: ([value highest SEP group – value lowest SEP group] / value highest SEP group) X 100. Odds ratio (OR) were reported for dichotomous outcome variables. Reference category for OR: Highest SEP group. *Relative differences >10% in intakes, or p<0.05 for OR.

Differences reported are for the lowest socioeconomic group relative to the highest group (i.e.: ‘-’ refers to lower intakes among the lower SEP group relative to the highest SEP group (or higher intakes among the highest SEP group relative to the lowest SEP group); ‘+‘ refers to higher intakes among the lower SEP group relative to the lowest SEP group (or lower intakes among the highest SEP group relative to the lowest SEP group). d: per day; pd: previous day.
